# Bacteria and bacterial envelope components enhance mammalian reovirus thermostability

**DOI:** 10.1371/journal.ppat.1006768

**Published:** 2017-12-06

**Authors:** Angela K. Berger, Hong Yi, Daniel B. Kearns, Bernardo A. Mainou

**Affiliations:** 1 Department of Pediatrics, Emory University School of Medicine, Atlanta, Georgia, United States of America; 2 Children’s Healthcare of Atlanta, Atlanta, Georgia, United States of America; 3 Robert P. Apkarian Integrated Electron Microscopy Core, Emory University, Atlanta, Georgia, United States of America; 4 Department of Biology, Indiana University, Bloomington, Indiana, United States of America; Cornell University, UNITED STATES

## Abstract

Enteric viruses encounter diverse environments as they migrate through the gastrointestinal tract to infect their hosts. The interaction of eukaryotic viruses with members of the host microbiota can greatly impact various aspects of virus biology, including the efficiency with which viruses can infect their hosts. Mammalian orthoreovirus, a human enteric virus that infects most humans during childhood, is negatively affected by antibiotic treatment prior to infection. However, it is not known how components of the host microbiota affect reovirus infectivity. In this study, we show that reovirus virions directly interact with Gram positive and Gram negative bacteria. Reovirus interaction with bacterial cells conveys enhanced virion thermostability that translates into enhanced attachment and infection of cells following an environmental insult. Enhanced virion thermostability was also conveyed by bacterial envelope components lipopolysaccharide (LPS) and peptidoglycan (PG). Lipoteichoic acid and N-acetylglucosamine-containing polysaccharides enhanced virion stability in a serotype-dependent manner. LPS and PG also enhanced the thermostability of an intermediate reovirus particle (ISVP) that is associated with primary infection in the gut. Although LPS and PG alter reovirus thermostability, these bacterial envelope components did not affect reovirus utilization of its proteinaceous cellular receptor junctional adhesion molecule-A or cell entry kinetics. LPS and PG also did not affect the overall number of reovirus capsid proteins σ1 and σ3, suggesting their effect on virion thermostability is not mediated through altering the overall number of major capsid proteins on the virus. Incubation of reovirus with LPS and PG did not significantly affect the neutralizing efficiency of reovirus-specific antibodies. These data suggest that bacteria enhance reovirus infection of the intestinal tract by enhancing the thermal stability of the reovirus particle at a variety of temperatures through interactions between the viral particle and bacterial envelope components.

## Author summary

Enteric viruses are exposed to diverse environments during their replication cycle. They must be stable enough to endure outside their host, persist through changing pH and proteolytic environments encountered through passage via digestive and gastrointestinal tracts, and pliable to undergo disassembly during infection of host cells. Some enteric viruses have evolved to interact with and use members of the host microbiota to achieve optimal virion stability to infect their host. In this study, we show that the enteric mammalian reovirus (reovirus) interacts with bacteria. The interaction of the virus with bacteria or bacterial envelope components results in enhanced virion stability, which translates into enhanced reovirus infectivity following an environmental stress. The interaction of reovirus with bacterial envelope components does not alter receptor utilization, overall infection kinetics, or affect the anti-viral effects of reovirus-specific antibodies. Together, we show that reovirus has evolved to use Gram positive and Gram negative bacteria to provide enhanced virion stability at a variety of temperatures.

## Introduction

Enteric viruses inhabit diverse environments and must achieve an equilibrium that permits environmental stability while allowing facile disassembly of the viral particle during infection of host cells. Enteric virus infection generally begins with the ingestion of contaminated fecal material via oral routes. Ingested viruses must transit through the enzyme-rich and low pH [1] environment of the stomach before reaching the gastrointestinal tract. In the gut, enteric viruses must navigate through an alkaline pH environment that is also abundant in proteases, glycoproteins (e.g. mucin), and a diverse microbial community [2, 3] to reach host epithelial or M cells [4]. Once inside host cells, viruses replicate and disseminate to secondary sites of infection and eventually are shed into the intestinal lumen. Enteric viruses have evolved a variety of mechanisms to successfully traverse through changing environments, infect the host, replicate, and disseminate, including the ability to use changes in pH, enteric enzymes, and commensal microorganisms.

Nonfusogenic mammalian orthoreovirus (reovirus) is a non-enveloped, segmented dsRNA virus, of the *Reoviridae* family that is formed by two concentric protein shells [5]. The *Reoviridae* include rotavirus, a leading causative agent of diarrhea in infants [6], and the arthropod-transmitted blue tongue virus, an important disease-causing agent in ruminants [7]. Reoviruses infect most mammals and although most humans are infected during childhood, infection seldom results in disease [5, 8, 9]. Reovirus generally infects humans via respiratory and intestinal tracts and was isolated from the stools of healthy and sick children [5, 10, 11]. Reovirus infection of the gut alters intestinal immune homeostasis, which can affect tolerance to dietary antigens like gluten [12]. Within the gut, reovirus infects the intestinal epithelium and is taken up and transported by intestinal M cells to underlying Peyer’s patches where replication and dissemination occur [4, 13–16]. Dissemination from the intestine takes place via hematogenous and neural routes [13, 14, 17, 18]. Subsequent infection of intestinal epithelial cells is thought to be an important source of virus shedding into the intestinal lumen [4, 19].

Reovirus attaches to cells through a strength adhesion mechanism in which the virus weakly binds to cell surface glycans [20, 21] followed by high affinity binding to junctional adhesion molecule-A (JAM-A) via the viral attachment fiber σ1 [22–24]. The Nogo-1 receptor (NgR1) is also important for infection of a subset of neuronal cells [25]. Following receptor engagement, reovirus is internalized via receptor-mediated endocytosis and traverses to late endosomes using microtubules [26–32]. In late endosomes, virions undergo stepwise proteolytic disassembly using host cysteine cathepsin proteases to form infectious subvirion particles (ISVPs) [33–35]. ISVP formation results in removal of outer capsid protein σ3 and cleavage of outer capsid protein μ1 [34–36]. During oral infection, digestive proteases within the intestine cleave reovirus virions into ISVPs [37]. ISVPs generated by different proteases, whether *in vitro*, intracellularly, or in the intestinal lumen are biochemically and functionally indistinguishable. During ISVP formation in endosomes, fragments that result from the cleavage of μ1 promotes endosomal penetration and release of the transcriptionally active viral core into the cytoplasm [34–36]. While several host and viral mediators of cell entry have been identified, the role of intestinal lumen components in reovirus infection are poorly understood.

In mice, clearance of intestinal bacteria through the administration of antibiotics prior to oral reovirus infection results in decreased infection and viral-associated pathology of the intestines [38]. Rotavirus infection and dissemination from infected animals is also impaired by treatment of mice with antibiotics prior to infection [39]. Two enteric viruses, poliovirus [38] and norovirus [40], as well as the retrovirus mouse mammary tumor virus (MMTV) [41], utilize different aspects of enteric commensal bacteria to successfully infect their hosts. Poliovirus interacts with bacterial lipopolysaccharides (LPS) through its VP1 capsid protein [42]. The interaction of VP1 with LPS promotes virion stability and enhances binding to the cellular poliovirus receptor (PVR) [42]. Norovirus uses histo-blood group antigen (HBGA) secreted by commensal bacteria to infect B cells [40]. LPS bound to the MMTV virion activates Toll-like receptor (TLR) signaling in the gut, creating an immunosuppressive microenvironment that allows immunological tolerance to MMTV antigens, thus allowing for MMTV persistence to be established [41]. Although intestinal bacteria affect the efficiency of reovirus infection in the gut, it is unclear how bacteria or bacterial envelope components affect reovirus infectivity.

In this study, we show that reovirus interacts with Gram positive and Gram negative bacteria. Incubation of reovirus with either Gram positive or Gram negative bacteria enhances the thermostability of virions, which translates into increased attachment and infection of eukaryotic cells following an environmental insult. The effect on enhanced virion thermostability is conveyed in part by bacterial LPS and peptidoglycan (PG). Incubation of reovirus virions or ISVPs at various temperatures with either LPS or PG enhance virion thermostability, attachment, and infection of eukaryotic cells. N-acetylglucosamine-free polysaccharide and mucin, but not sucrose, also enhance virion stability in a serotype-independent manner. Reovirus infection in the presence of LPS or PG does not alter reovirus receptor utilization, overall cell entry kinetics, or the efficiency of antibody-mediated neutralization. Together, data presented in this study show for the first time that an interaction of reovirus with bacteria through bacterial envelope components enhance virion thermostability.

## Results

### Gram negative and Gram positive bacteria increase reovirus attachment and infectivity following heat shock

**Fig 1:**
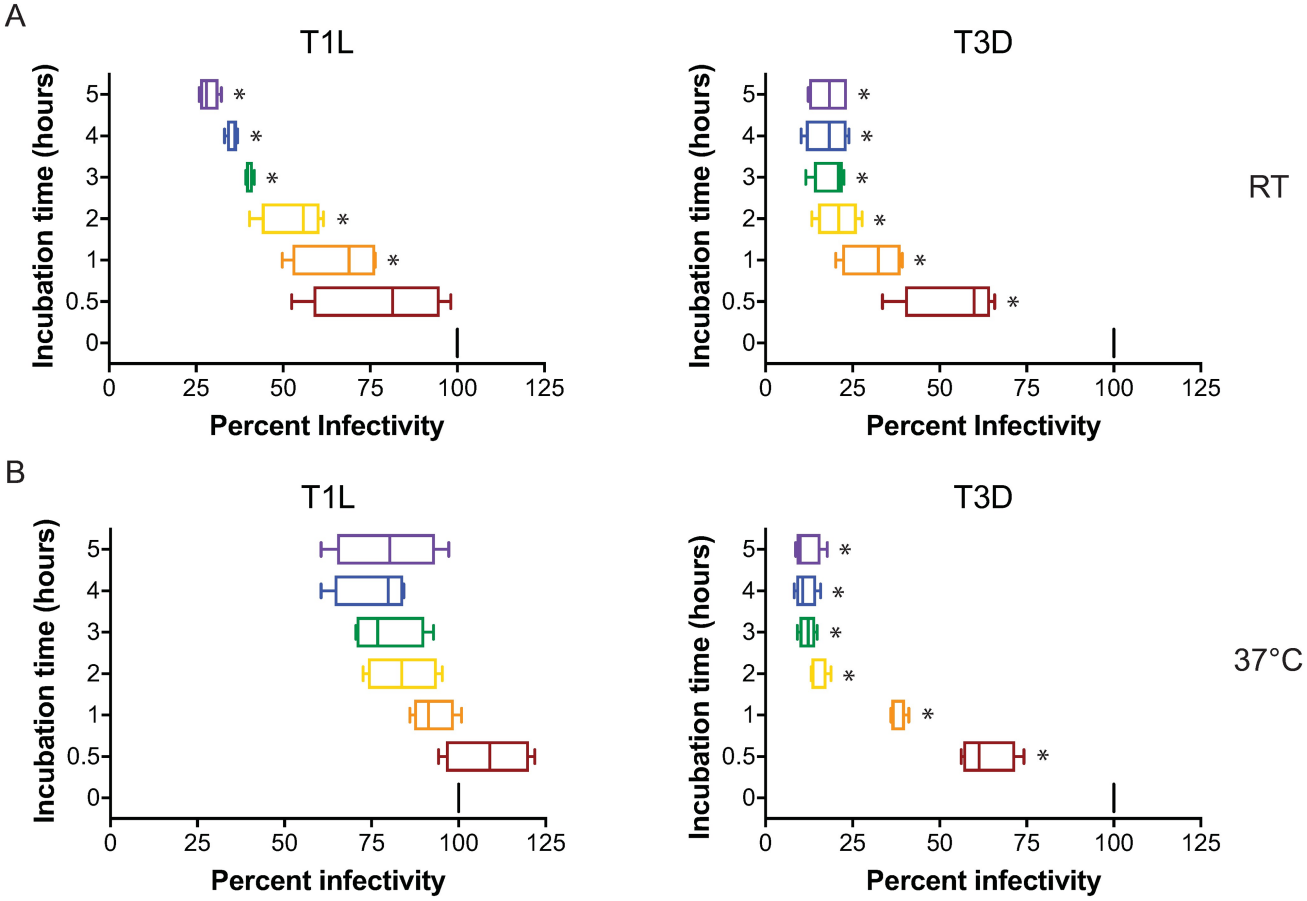
Thermostability of reovirus T1L and T3D. Reovirus strains T1L and T3D were incubated in PBS at (A) room temperature or (B) 37°C for indicated times, adsorbed on HeLa cells at an MOI of 5×10^3^ particles/cell, and assessed for infectivity in HeLa cells 18 hpi by indirect immunofluorescence. Results are expressed as box and whisker plots of percent infectivity (normalized to 0 min) for quadruplicate independent experiments. *, *P* < 0.0005 in comparison to 0 h by one-way ANOVA with Dunnett’s multiple-comparison test.

Antibiotic treatment of mice prior to oral inoculation with poliovirus or reovirus decreases infectivity and intestinal pathology compared to untreated animals [38]. Poliovirus binding of bacterial polysaccharides increases viral thermal stability but it is not known if bacterial polysaccharides affect reovirus stability [38, 42]. To assess the thermostability of reovirus prototypical strains Type 1 Lang (T1L) and Type 3 Dearing (T3D), reovirus virions and infectious subvirion particles (ISVPs) were incubated for 0–5 h at room temperature (23°C, Fig 1A), 28°C (S1A Fig), or 37°C (Fig 1B), adsorbed on HeLa cells, which support reovirus infection [30, 31, 43, 44], and scored for infectivity by indirect immunofluorescence using reovirus-specific antisera. The thermostability of T1L virions was greater than that of T3D at all temperatures tested. At room temperature, T1L virions retained more than 50% of its infectivity after 2 h, whereas T3D lost over 50% of its infectivity by 1 h and ~80% of its infectivity by 2 h. Incubation of T1L or T3D at 28°C and 37°C paradoxically did not increase the rate at which viruses lose infectivity, although at all temperatures tested T3D had lost over 75% of its infectivity by 2 h. Interestingly, other groups have observed gradual infectivity increases between 33°C and 37°C, with peak infectivity observed at 37°C [45]. Similar to that observed with virions, the thermostability of T1L ISVPs was greater than T3D ISVPs (S1B, S1C and S1D Fig). At all temperatures tested, T1L ISVPs retained over 50% of their infectivity after 2 h. In contrast, T3D ISVPs lost over 50% of their infectivity by 1 h. In comparison to virions, ISVPs are less thermostable than virions. These data are concordant with previous studies showing T1L and reovirus strain Type 2 Jones (T2J) having greater thermostability than T3D [46], virions being more thermostable than ISVPs [45], and provide a temporal window to study the effects of bacteria and bacterial polysaccharides on reovirus thermostability and infection.

**Fig 2:**
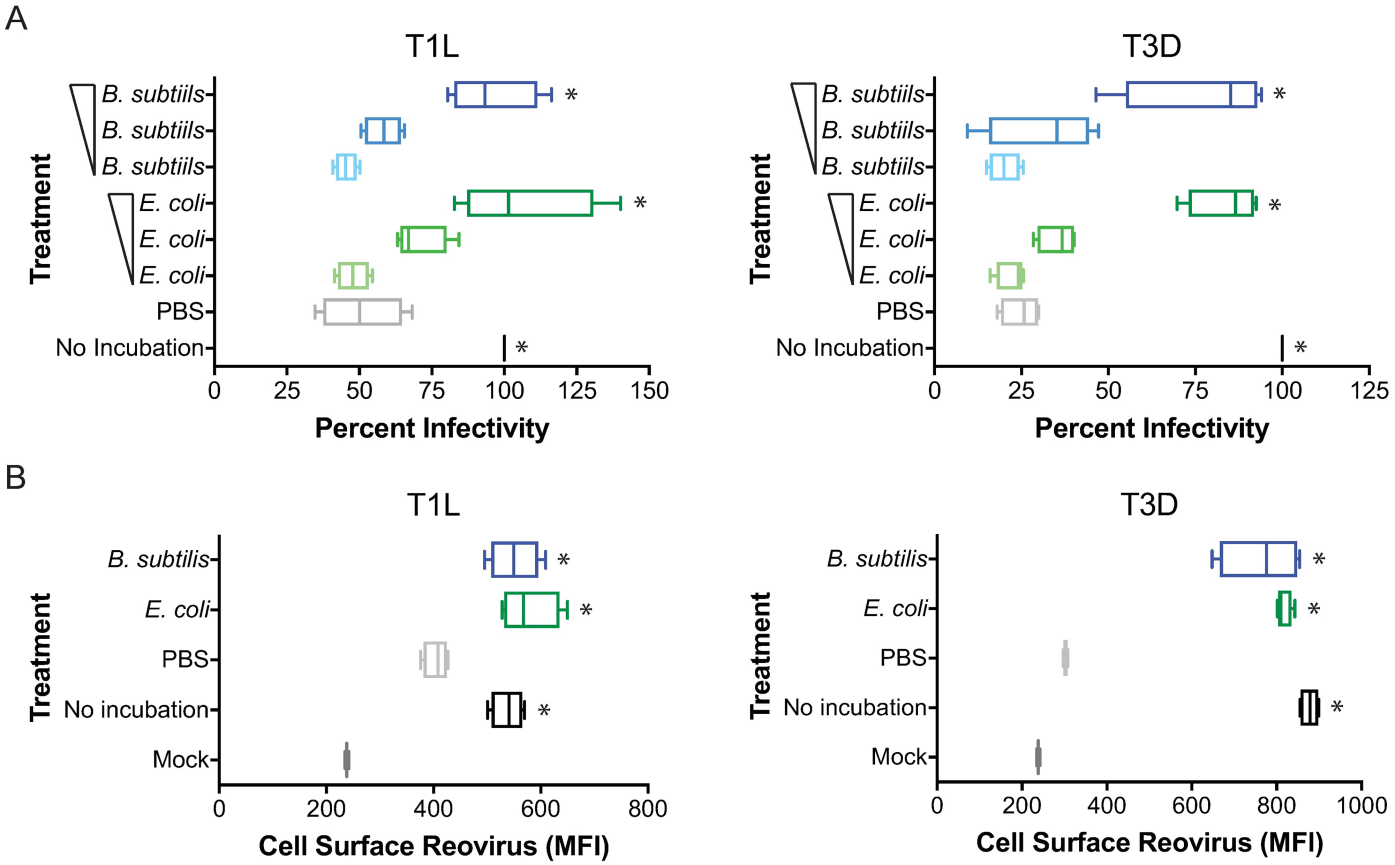
Gram positive and Gram negative bacteria positively affect reovirus thermostability. (A) Reovirus T1L and T3D were not incubated, incubated with PBS or increasing concentrations of *B*. *subtilis* or *E*. *coli* for 2 h at room temperature. HeLa cells were adsorbed with reovirus at an MOI of 5×10^3^ particles/cell and assessed for infectivity in HeLa cells 18 hpi by indirect immunofluorescence. Results are expressed as box and whisker plots of percent infectivity (normalized to no incubation) for quadruplicate independent experiments. (B) A488-labeled reovirus T1L and T3D were not incubated, incubated with PBS or *B*. *subtilis* or *E*. *coli* for 2 h at room temperature and 5×10^3^ particles/cell were assessed for attachment to HeLa cells by flowcytometry. Results are expressed as box and whisker plots of cell surface reovirus as mean fluorescence intensity (MFI) for quadruplicate independent experiments. *, *P* < 0.0005 in comparison to PBS by one-way ANOVA with Dunnett’s multiple-comparison test.

To determine if bacteria affect reovirus infectivity and thermostability, reovirus T1L and T3D were not incubated or incubated in PBS, or in the presence of increasing concentrations of Gram positive *B*. *subtilis* or Gram negative *E*. *coli*, for 2 h at room temperature, and assessed for infectivity in HeLa cells by indirect immunofluorescence using reovirus-specific antisera (Fig 2A). Incubation of reovirus in PBS substantially impaired viral infectivity, with T1L infectivity decreasing by 50% and T3D by over 75%. Incubation of T1L or T3D with either *B*. *subtilis* or *E*. *coli* prevented loss of infectivity in a concentration-dependent manner to ~100% infectivity with T1L and ~80% infectivity with T3D when virus was incubated with 9.50 × 10^8^ CFUs of *B*. *subtilis* or 2.35 × 10^9^ CFUs of *E*. *coli* (which amounts to ~1:1 ratio of particles to CFUs). Although T1L infectivity in the presence of bacteria were higher than those of T3D, the magnitude of the protection provided by *B*. *subtilis* or *E*. *coli* on either virus were almost identical. These data suggest both Gram positive and Gram negative bacteria can positively affect reovirus thermostability.

To pinpoint the step in the reovirus replication cycle affected by environmental insult and incubation of reovirus with bacteria, we first assessed if attachment to cells was affected. Alexa 488 (A488)-labeled T1L and T3D were not incubated or incubated with PBS, *B*. *subtilis*, or *E*. *coli* for 2 h at room temperature, and assessed for viral attachment to HeLa cells by flowcytometry (Fig 2B). Incubation of T1L and T3D in PBS decreased viral attachment to cells, with T3D attachment being more significantly impaired than T1L. Incubation of T1L or T3D with *B*. *subtilis* or *E*. *coli* restored viral attachment to cells to similar levels than those observed with virus that had not been incubated with bacteria. The effects on cell attachment following incubation of T1L or T3D with bacteria phenocopy those observed with infectivity. These data suggest the effects of environmental assault on reovirus alter the viral particle in a way that affect attachment and subsequent infection of cells. These data also indicate that incubation of virus with *B*. *subtilis* or *E*.*coli* prevent reovirus loss of infectivity following an environmental insult.

### Reovirus directly associates with bacteria

**Fig 3:**
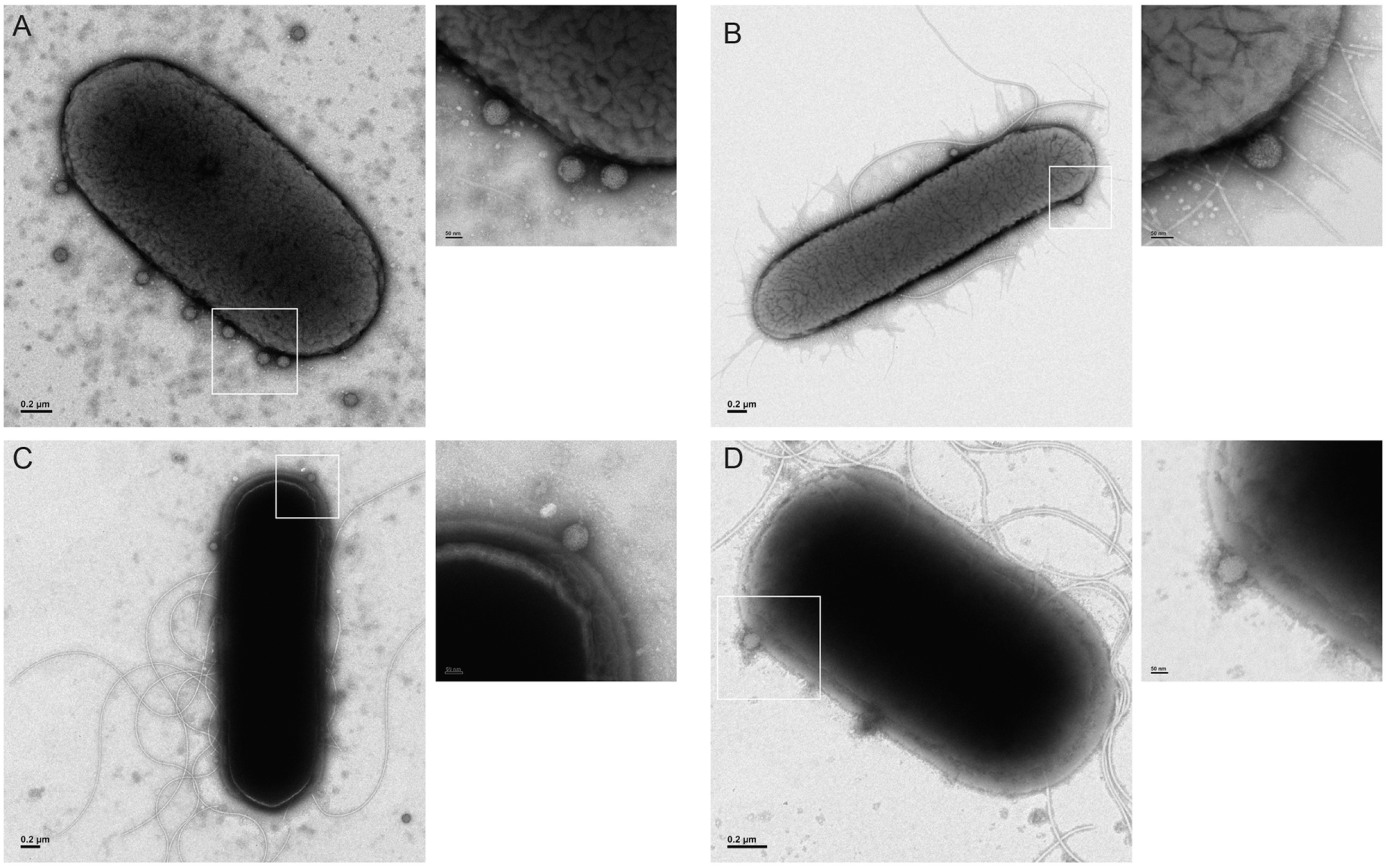
Reovirus associates with Gram negative and Gram positive bacteria. Reovirus T1L (A, C) or T3D (B, D) was incubated with *E*. *coli* (A, B) or *B*. *subtilis* (C, D) for 2 h at room temperature. Cells were stained with sodium phosphotungstic acid (PTA) and imaged by electron microscopy. Scale bars 0.2 μm in whole cell images, 50 nm in insets.

To define how bacteria influence reovirus infectivity and determine why infectivity decreases following an environmental insult, the integrity and structure of viral particles were assessed following incubation in PBS for 2 h at room temperature by negative stain transmission electron microscopy (EM). To assess if reovirus virions interact with Gram positive or Gram negative bacteria, T1L and T3D virions were incubated with *B*. *subtilis* or *E*. *coli* and imaged by negative stain EM (Fig 3). T1L and T3D virions were observed to be associated with *E*. *coli* (Fig 3A and 3B) and *B*. *subtilis* (Fig 3C and 3D) cells, with virions appearing to directly bind to the envelope of both *B*. *subtilis* and *E*. *coli* without causing the aggregation of viral particles. Virions were also observed in the vicinity of *E*. *coli* pili, but did not seem to directly associate with these structures (Fig 3B). These data show that reovirus can associate with Gram positive and Gram negative bacteria and suggest that the effects of bacteria on reovirus thermostability and infectivity are mediated through the direct interaction of bacterial outer envelope components and virions.

### Bacterial outer envelope components protect reovirus from environmental assault

**Fig 4:**
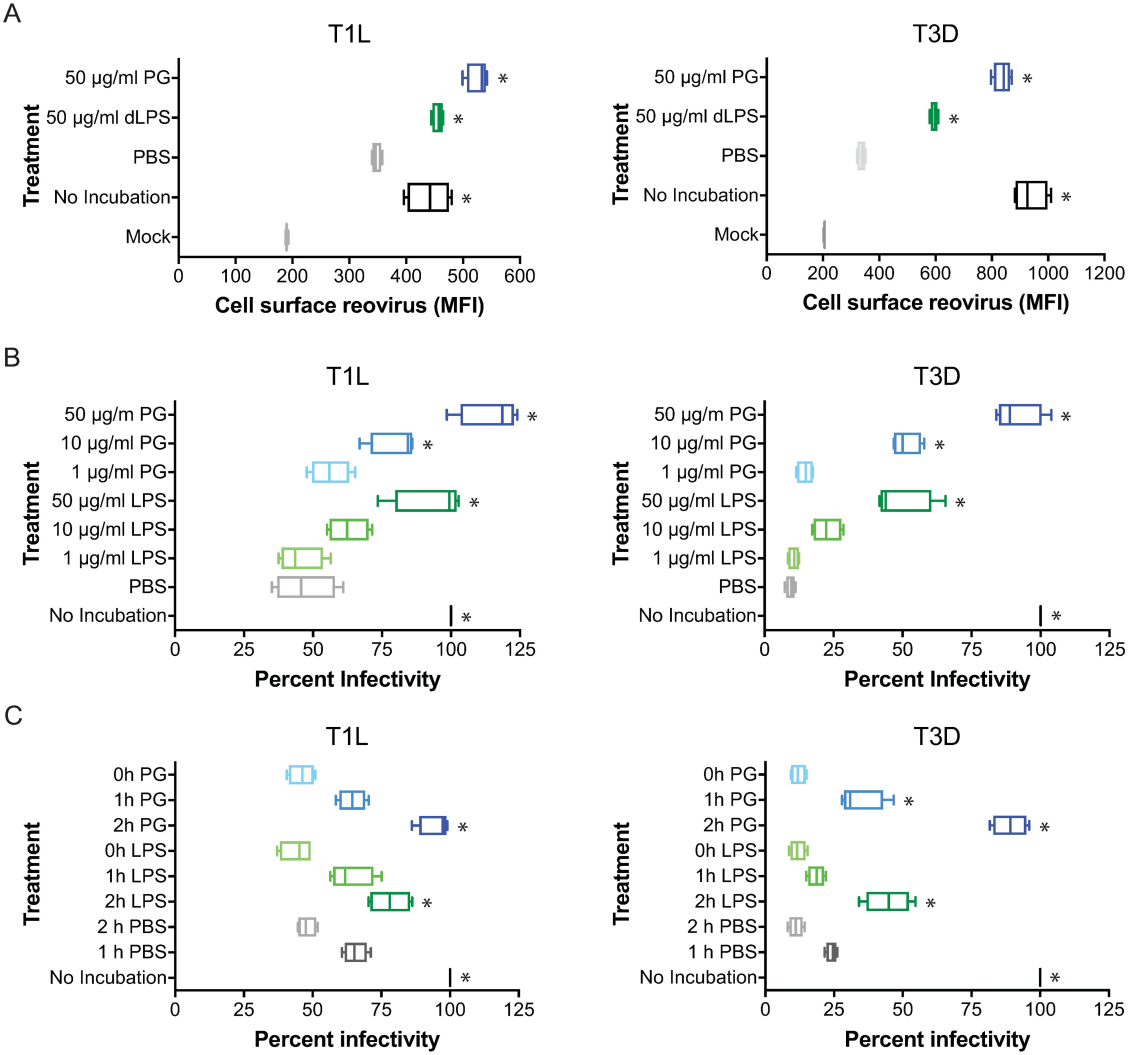
Lipopolysaccharide and peptidoglycan protect reovirus from loss of attachment and infectivity. (A, B) Reovirus T1L and T3D were not incubated, incubated with PBS, detoxified LPS (dLPS), LPS, or PG for 2 h at room temperature. (C) Reovirus T1L and T3D were not incubated or incubated with PBS for 2 h at room temperature. 50 μg/ml LPS or 50 μg/ml PG were added at indicated times during 2 h incubation. (A) HeLa cells were adsorbed with A633-labeled reovirus at an MOI of 5×10^3^ particles/cell and assessed for reovirus attachment by flowcytometry. Results are expressed as box and whisker plots of cell surface reovirus as MFI for quadruplicate independent experiments. (B, C) HeLa cells were adsorbed with reovirus at an MOI of 5×10^3^ particles/cell, incubated for 18 h, and scored for infectivity by indirect immunofluorescence. Results are expressed as box and whisker plots of percent infectivity (normalized to no incubation) for quadruplicate independent experiments. *, *P* < 0.0005 in comparison to PBS by one-way ANOVA with Dunnett’s multiple-comparison test.

Bacterial envelopes are composed of two main cell surface components, peptidoglycan (PG) in Gram positive bacteria and lipopolysaccharide (LPS) in Gram negative bacteria, although Gram negative bacteria also contain PG [47]. Poliovirus directly binds bacterial LPS through its viral capsid protein VP1 [38, 42]. To determine if bacterial outer envelope components can affect reovirus thermostability, reovirus T1L and T3D were not incubated or incubated with PBS, purified detoxified LPS (dLPS), which lacks lipid A, from *E*. *coli*, or PG from *B*. *subtilis* for 2 h at room temperature, and assessed for attachment to HeLa cells by flowcytometry (Fig 4A). Incubation of T1L or T3D with 50 μg/ml PG resulted in a slight increase in attachment to cells (T1L) or almost similar levels (T3D) to those observed with non-incubated virus. Incubation of T1L with 50 μg/ml dLPS restored attachment to cells to similar levels than non-incubated virus, but only partially restored attachment of T3D to cells. Although T3D attachment is not restored to the same levels as those observed with non-incubated virus, the magnitude of the protection provided by dLPS (1.8x) and PG (2.5x) is slightly greater than that observed with T1L (dLPS 1.3x) and PG (1.5x). These data suggest that T1L and T3D interact with both LPS and PG and that this interaction is sufficient to preserve viral attachment to target cells that is lost following an environmental insult.

To determine the effect of LPS or PG on reovirus infectivity, T1L and T3D were not incubated, incubated with PBS, or increasing concentrations of LPS or PG for 2 hours at room temperature, and assessed for infectivity on HeLa cells by indirect immunofluorescence using reovirus-specific antisera (Fig 4B). Incubation of T1L or T3D with PG resulted in a dose-dependent increase in infectivity that parallels that observed when the virions were incubated with *B*. *subtilis*. T1L incubated in the presence of 50 μg/ml of PG resulted in a slight increase in infectivity beyond what was observed with virus that had not been incubated. Incubation of T1L or T3D with LPS resulted in a dose-dependent increase in infectivity that mirrors observations made when either virus was incubated with *E*. *coli*, although incubation with bacteria had a more robust effect on T3D infectivity than LPS alone. Whereas full infectivity was restored when T1L was incubated with 50 μg/ml of LPS, incubation of T3D with LPS restored infectivity to slightly less than 50% compared to non-incubated virus, although the magnitude of the change was greater than that observed with T1L. The effects of the bacterial envelope components on T1L and T3D infectivity phenocopy the effects observed when assessing viral attachment to cells. These data suggest that LPS and PG alter reovirus thermostability by preventing an environmental-mediated change in the virus. These data also suggest that other components of Gram negative bacteria beyond LPS affect T3D virion stability.

To define the temporal window in which LPS and PG can affect reovirus thermostability, T1L and T3D were not incubated, incubated at room temperature with PBS for 1 or 2 h, or incubated with PBS with LPS and PG being added at 1 h intervals (Fig 4C). As observed previously, T3D infectivity decreased with faster kinetics than T1L when incubated in PBS. Addition of LPS and PG after the 2 h incubation did not enhance virion stability of T1L or T3D. Addition of LPS and PG after 1 h of incubation had an intermediate effect on virion stability compared to virus that had been incubated with bacterial envelope components for the entire time course. These data suggest that the effects on virion stability cannot be reversed by LPS and PG but can be arrested following thermal stress.

To assess if the effects on viral thermostability are cell line specific, T1L and T3D were not incubated, incubated with PBS, dLPS, LPS, or PG for 2 h at room temperature, and assessed for attachment (S2A Fig) and infectivity (S2B Fig) on colonic adenocarcinoma (Caco2) cells. Similar to that observed in HeLa cells, LPS and PG restored T1L and T3D attachment and infectivity, with PG having a more robust effect on both T1L and T3D than LPS. Although LPS only restored T3D infectivity to ~50% of that from non-incubated virus, the magnitude of the protection provided was significantly larger than that observed with T1L. Also, the infectivity of both T1L and T3D were slightly enhanced by the highest concentration of PG. These data show that the effect of bacterial envelope components on viral thermostability is not dependent on the cells used to score for attachment or infectivity.

### Bacteria-associated reovirus is delivered to eukaryotic cells

**Fig 5:**
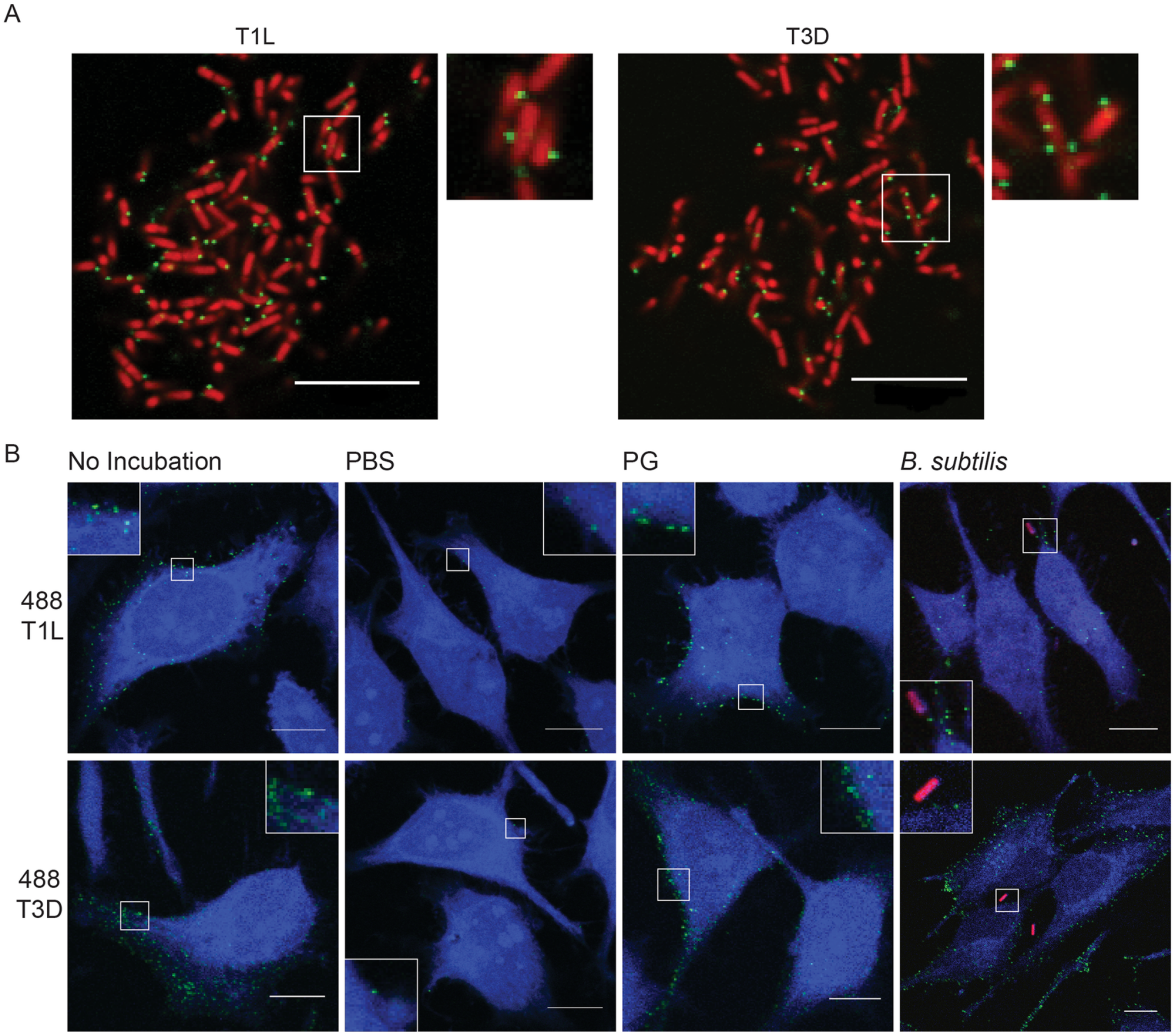
Association of reovirus with bacteria enhances attachment to eukaryotic cells. (A) A488-labeled T1L and T3D reovirus were incubated with mCherry-expressing *B*. *subtilis* for 2 h at room temperature and imaged by confocal microscopy. (B) A488-labeled T1L and T3D reovirus were not incubated or incubated with PBS, 20 μg/ml PG, or mCherry-expressing *B*. *subtilis* for 2 h at room temperature and adsorbed to HeLa cells for 1 h. Cells were stained with CellTracker Blue and imaged by confocal microscopy. Scale bar, 10 μm.

To visualize the effect of PG and *B*. *subtilis* on reovirus infection, A488-labeled T1L and T3D reovirus were not incubated, incubated with PBS, mCherry-expressing *B*. *subtilis*, or PG for 2 h at room temperature, adsorbed for 1 h on HeLa cells, and imaged by confocal microscopy (Fig 5). Similar to that observed by electron microscopy, reovirus T1L and T3D associate with *B*. *subtilis* cells (Fig 5A). Incubation of reovirus in PBS resulted in a substantial decrease in the number of virions associated with HeLa cells and number of cells with bound virus as compared to non-incubated virus (Fig 5B). Incubation of T1L or T3D with PG or *B*. *subtilis* resulted in a significant number of virions associated with HeLa cells. The levels of reovirus associated with HeLa cells appeared similar between non-incubated virus and PG- or *B*. *subtilis*-incubated virus. Also, more virions were detected on cells infected with T3D than T1L, which corroborates flow cytometry attachment data showing that T3D can attach to HeLa cells more efficiently than T1L. Interestingly, following adsorption of *B*. *subtilis*-containing reovirus, very few *B*. *subtilis* cells were observed. It is possible UV-inactivation of *B*. *subtilis* affects the ability of the bacteria to attach to HeLa cells or that HeLa cells are not conducive for *B*. *subtilis* attachment. These data suggest that reovirus loss of infectivity following environmental assault is linked to a loss in the ability of the virus to attach to eukaryotic cells. These data also show that incubation of reovirus with *B*. *subtilis* or PG does not cause the aggregation of viral particles at the cell surface.

### Reovirus thermostability is differentially impacted by polysaccharides and small molecules

**Fig 6:**
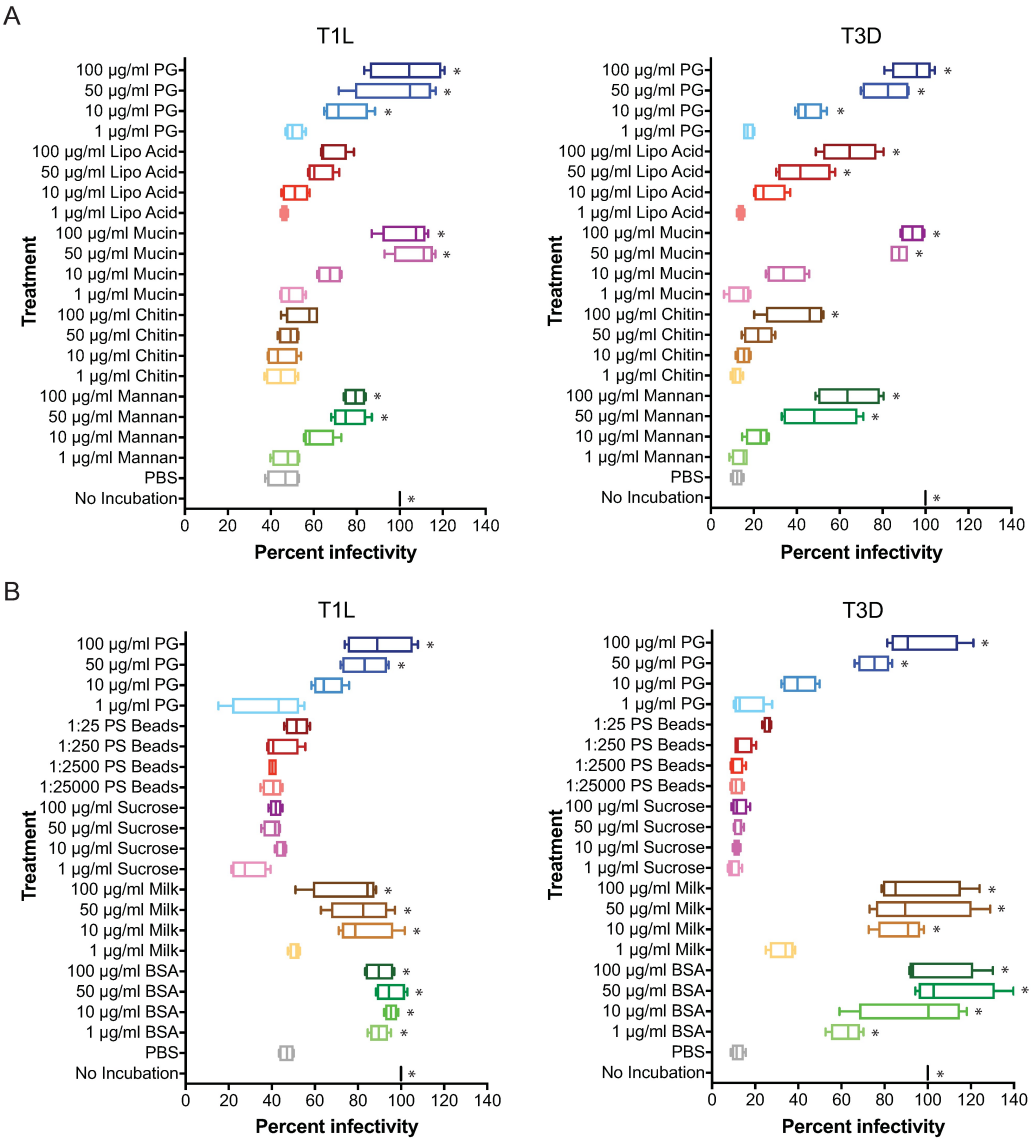
Composition of polysaccharides differentially affect reovirus thermostability. (A) Reovirus T1L and T3D were not incubated, incubated with PBS or increasing concentrations of mannan, chitin, mucin, lipoteichoic acid (Lipo Acid), or PG for 2 h at room temperature. (B) Reovirus T1L and T3D were not incubated, incubated with PBS or increasing concentrations of BSA, milk, sucrose, polystyrene beads (PS Beads), or PG for 2 h at room temperature. PS beads concentration is shown as the ratio of virions to beads. (A, B) HeLa cells were adsorbed with reovirus at an MOI of 5×10^3^ particles/cell, incubated for 18 h, and scored for infectivity by indirect immunofluorescence. Results are expressed as box and whisker plots of percent infectivity (normalized to no incubation) for quadruplicate independent experiments. *, *P* ≤ 0.0005 in comparison to PBS by one-way ANOVA with Dunnett’s multiple-comparison test.

The Gram positive bacterial envelope is composed of a thick layer of PG, lipoteichoic acid (lipo Acid), teichoic acid, and bacterial proteins [47]. PG is composed of alternating N-acetylglucosamine (GlcNAc) and N-acetylmuramic acid (NAM) sugars linked to a short amino acid chain [47]. To determine the contribution of GlcNAc, bacterial envelope carbohydrates, and mucin, a highly glycosylated molecule that lines the gastrointestinal tract [3], to reovirus thermostability, T1L and T3D were not incubated or incubated with PBS or increasing concentrations of mannan (GlcNAc-free polysaccharide), chitin (GlcNAc-containing polysaccharide), mucin, lipo acid, or PG for 2h at room temperature, and assessed for infectivity on HeLa cells (Fig 6A). Mannan, mucin, and PG enhanced the thermostability of both T1L and T3D in a concentration-dependent manner, although mannan did not enhance virion thermostability as potently as mucin or PG. In contrast, chitin and lipo acid did not significantly enhance T1L thermostability. Lipo acid enhanced T3D thermostability to similar levels as mannan and chitin slightly enhanced T3D thermostability only at the highest concentration tested. These data suggest that the polysaccharide requirements that convey enhanced reovirus thermostability differ between T1L and T3D. Whereas neither GlcNAc nor lipo acid enhance T1L virion stability, GlcNAc has a minor contribution to enhancing T3D thermostability and lipo acid significantly enhances the T3D thermostability.

To determine if complex molecules or the presence of macromolecular objects affect reovirus thermostability, T1L and T3D were not incubated or incubated in PBS or increasing concentrations of bovine serum albumin (BSA), powdered milk (milk), sucrose (disaccharide composed of glucose and fructose), polystyrene (PS) beads, or PG for 2 h at room temperature, and assessed for infectivity on HeLa cells (Fig 6B). BSA and milk enhanced both T1L and T3D thermostability to similar levels as PG. In contrast, sucrose and PS beads did not enhance reovirus thermostability. These data indicate that while bacterial envelope components can enhance virion stability, BSA, which is stabilizes proteins through hydrophobic interactions [48, 49], and milk can also enhance the thermostability of both strains of reovirus. However, the composition of the polysaccharide also dictates if it will have an impact on virion stability. These data also indicate that macromolecular objects are not sufficient to enhance reovirus thermostability.

### LPS and PG do not alter JAM-A usage for reovirus attachment

**Fig 7:**
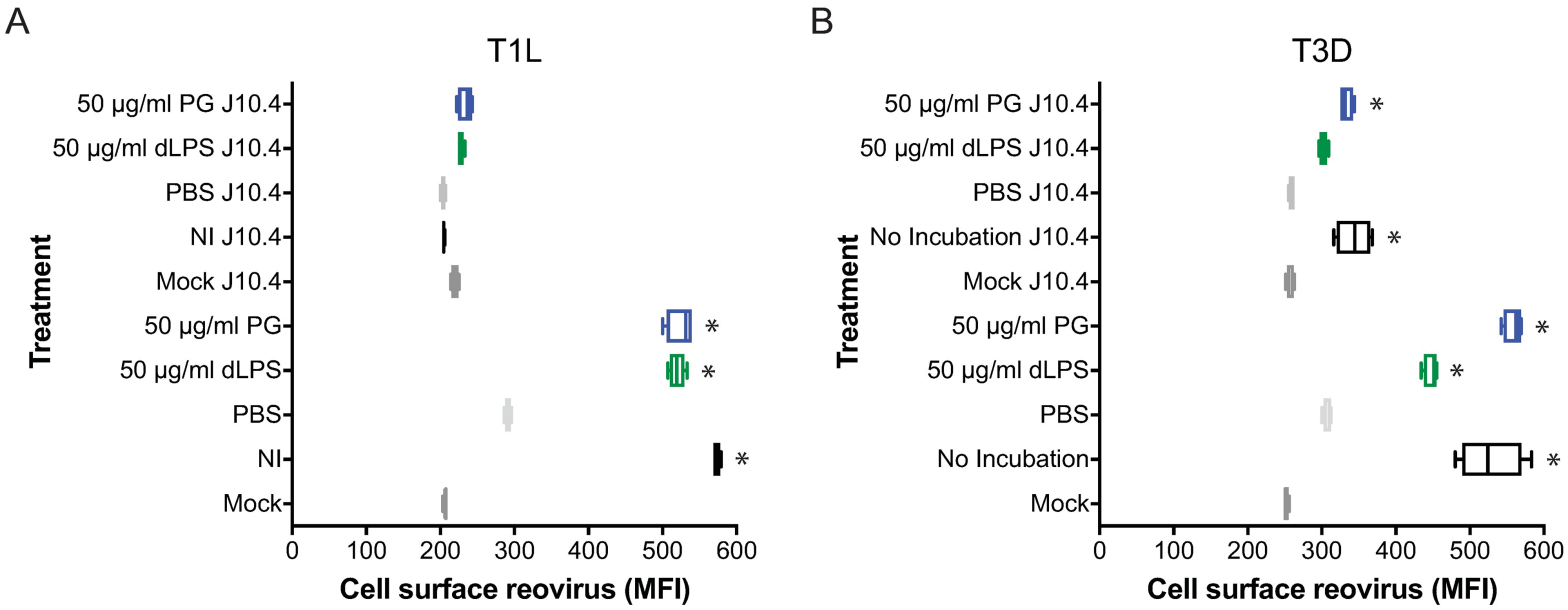
Lipopolysaccharide and peptidoglycan do not affect reovirus receptor utilization. A488-labeled (A) T1L and (B) T3D reovirus were not incubated (NI) or incubated with PBS, 50 μg/ml detoxified LPS (dLPS), or 50 μg/ml PG for 2 h at room temperature. HeLa cells were incubated with 10 μg/ml JAM-A specific antibody for 1 h at 4°C prior to adsorption with reovirus at an MOI of 5×10^3^ particles/cell for 1 h at room temperature. Cell surface reovirus (MFI) was assessed by flow cytometry. *, *P* < 0.0005 compared to PBS incubated virus by one-way ANOVA with Dunnett’s multiple-comparison test.

Reovirus engages JAM-A on the surface of cells to initiate infection on most cells [22–24]. To determine if LPS or PG alter JAM-A-dependent reovirus attachment to cells, A488-labeled reovirus was not incubated, incubated with PBS, dLPS, or PG for 2 h at room temperature, adsorbed to HeLa cells that had been incubated with no antibody or JAM-A-specific antibody (J10.4), and scored for cell attachment by flowcytometry (Fig 7). Treatment of cells with JAM-A-specific antibody blocked attachment of T1L (Fig 7A) and T3D (Fig 7B) reovirus to cells regardless of the presence or absence of LPS or PG. The small increase in infectivity observed with non-incubated, LPS-, and PG-incubated T3D in the presence of JAM-A antibody is likely due to carbohydrate binding. In contrast, residual carbohydrate binding was not observed with T1L. T1L and T3D binding different cell-surface carbohydrates [21, 50]. The difference in residual attachment could reflect different levels of cell surface carbohydrate available to T1L and T3D. PBS-incubated T3D completely lost its ability to attach to cells, suggesting the loss of infectivity observed with T3D is due to the inability to engage cell surface carbohydrate and JAM-A. These data show that reovirus uses of JAM-A to attach to cells is not affected by LPS or PG.

### LPS and PG enhance the thermostability of infectious subvirion particles

**Fig 8:**
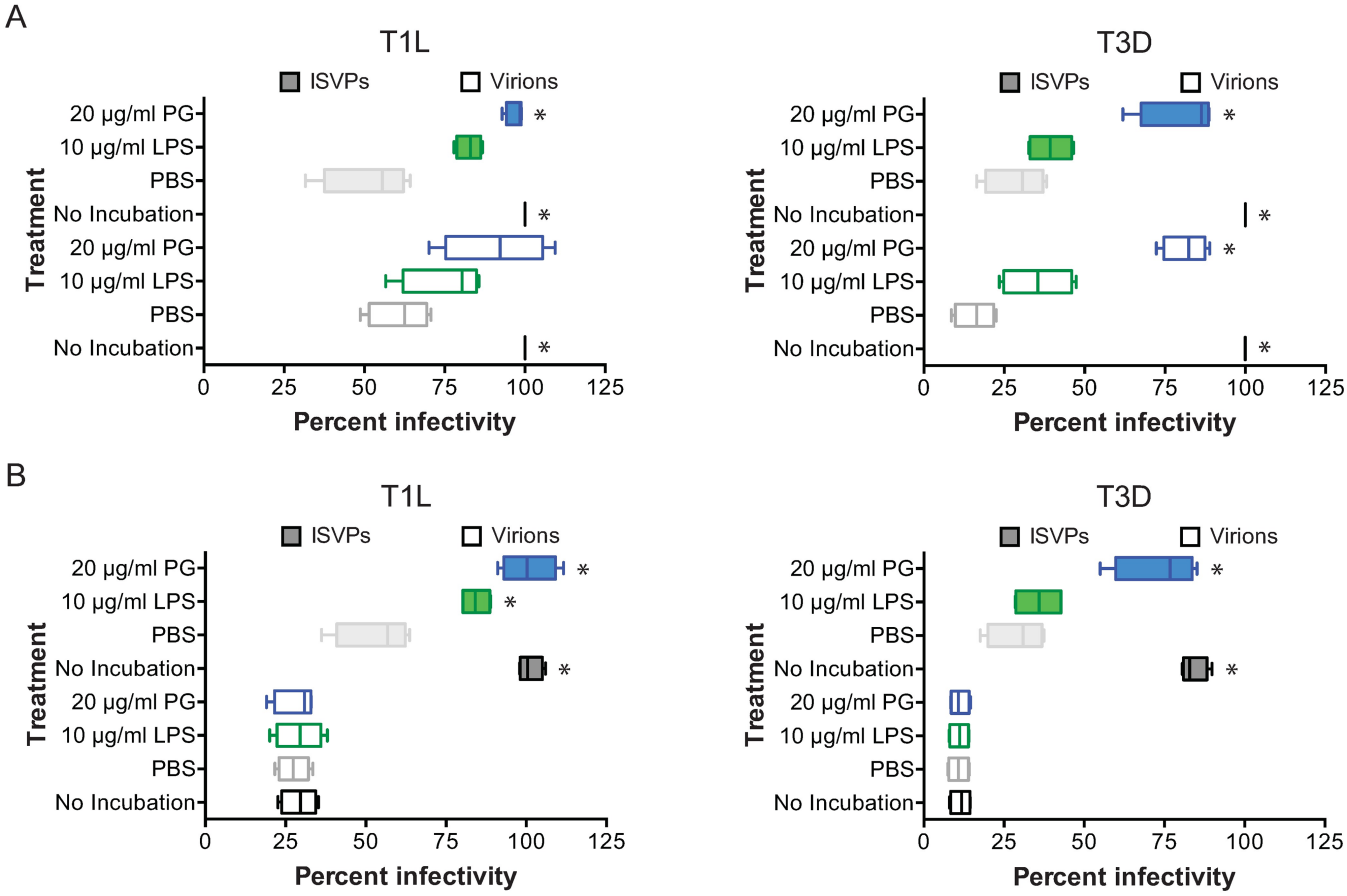
Lipopolysaccharide and peptidoglycan protect ISVPs from loss of infectivity but do not affect post cell entry steps. Reovirus T1L and T3D virions and ISVPs were not incubated or incubated with PBS, 10 μg/ml LPS, or 20 μg/ml PG for 2 h at room temperature. HeLa cells were (A) untreated or (B) treated with 20 mM ammonium chloride for 1 h prior to adsorption at an MOI of 5×10^3^ particles/cell (virions) or 1×10^3^ particles/cell (ISVPs). Cells were incubated for 18 h in media or 20 mM ammonium chloride and scored for infectivity by indirect immunofluorescence with reovirus-specific antiserum. Results are shown as box and whisker plots for quadruplicate independent experiments. *, *P* < 0.0005 compared to PBS incubated virus by one-way ANOVA with Dunnett’s multiple-comparison test.

During cell entry, reovirus undergoes acid-dependent stepwise proteolytic disassembly by cathepsin proteases following receptor-mediated endocytosis to form ISVPs [33–36]. ISVPs are also formed in the lumen of infected animals during oral inoculation and are capable of infecting cells in the gut [37, 51]. As such, it is possible that the reovirus population that associates with bacteria in the gut is a mixture of virions and ISVPs. To evaluate if LPS and PG have similar effects on ISVPs than virions, T1L virions and *in vitro*-generated ISVPs were not incubated, incubated with PBS, LPS, or PG for 2 h at room temperature, adsorbed on HeLa cells, and scored for infectivity by indirect immunofluorescence using reovirus-specific antisera (Fig 8A). T1L and T3D ISVPs lost infectivity when incubated in PBS, although T1L ISVPs are more thermostable than T3D ISVPs. Virions and ISVPs were protected from loss of infectivity by PG to similar levels. Similar to that observed with virions LPS enhanced T1L ISVP thermostability more than T3D ISVPs. These data suggest that LPS and PG can enhance the thermostability of both reovirus particle types that have been associated with infection in the gut. ISVPs lack structural protein σ3 [34–36], indicating that the effects of PG on viral stability and infectivity are likely not mediated by interactions with this viral protein. These data also suggest that the contact sites of LPS with T1L and T3D may be distinct, with the possibility that the σ3 protein of T3D could bridge the interaction between LPS and the virion.

Infection of cells by ISVPs takes place at or near the cell surface [26, 52]. As such, infection of cells by ISVPs bypasses the acid-dependent proteolytic processing required by virions [36]. To determine if LPS or PG affect ISVP’s ability to infect cells in an acid-independent manner, we assessed ISVP infectivity in the presence or absence of the weak base ammonium chloride (AC). T1L and T3D virions or ISVPs were not incubated, incubated with PBS, LPS, or PG for 2 h at room temperature, adsorbed on HeLa cells that had been untreated or treated with 20 mM AC, and scored for infectivity by indirect immunofluorescence using reovirus-specific antisera (Fig 8B). In untreated cells, virions and ISVPs had decreased infectivity when incubated in PBS as compared to non-incubated, LPS-, or PG-incubated reovirus (Fig 8A). In cells treated with AC, infectivity with virions was completely impaired regardless of the presence or absence of LPS or PG (Fig 8B). In contrast, infection of AC-treated cells with ISVPs was not impaired regardless of the presence or absence of LPS or PG. These data indicate that LPS and PG do not affect overall cell entry kinetics or infection of cells by ISVPs.

To determine if LPS or PG can affect reovirus infectivity of virions and ISVPs in the absence of thermal stress, T1L and T3D virions and ISVPs were not incubated or incubated with increasing concentrations of LPS or PG for 2 h at 4°C, and assessed for infectivity on HeLa cells (S3 Fig). The infectivity of T1L or T3D virions was not significantly affected by incubation with PBS, LPS, or PG (S3A Fig). Whereas the infectivity of T1L ISVPs was not impacted by incubation with PBS, LPS, or PG, the infectivity of T3D ISVPs was slightly enhanced by incubation with the highest concentration of PG (S3B Fig). These data show that virions and ISVPs are stable following incubation at 4°C and that LPS does not enhance infectivity of either virions or ISVPs in the absence of thermal stress. These data also suggest that although PG does not enhance the infectivity of T1L virions, T1L ISVPs, or T3D virions, it provides a slight enhancement to the infectivity of T3D ISVPs.

To determine how LPS and PG affect reovirus virions and ISVPs over a range of temperatures, T3D virions and ISVPS were not incubated or incubated with PBS, LPS, or PG for 2 h at room temperature, 28°C, or 37°C, and assessed for infectivity in HeLa cells (S4 Fig). As previously observed, the infectivity of virions and ISVPs was significantly impaired regardless of the temperature. Incubation of virions (S4A Fig) and ISVPs (S4B Fig) with PG yielded similar effects on infectivity regardless of the temperature. In contrast, the effect of LPS on virion and ISVP stability was greater at higher temperatures with peak enhancement observed at 37°C for virions and 28°C for ISVPs. These data suggest that although LPS and PG enhance reovirus virion stability, they likely do so by engaging different sites within the reovirus virion and have differing effects on the virus at changing temperatures.

### Cell entry kinetics are not altered by bacterial envelope components

**Fig 9:**
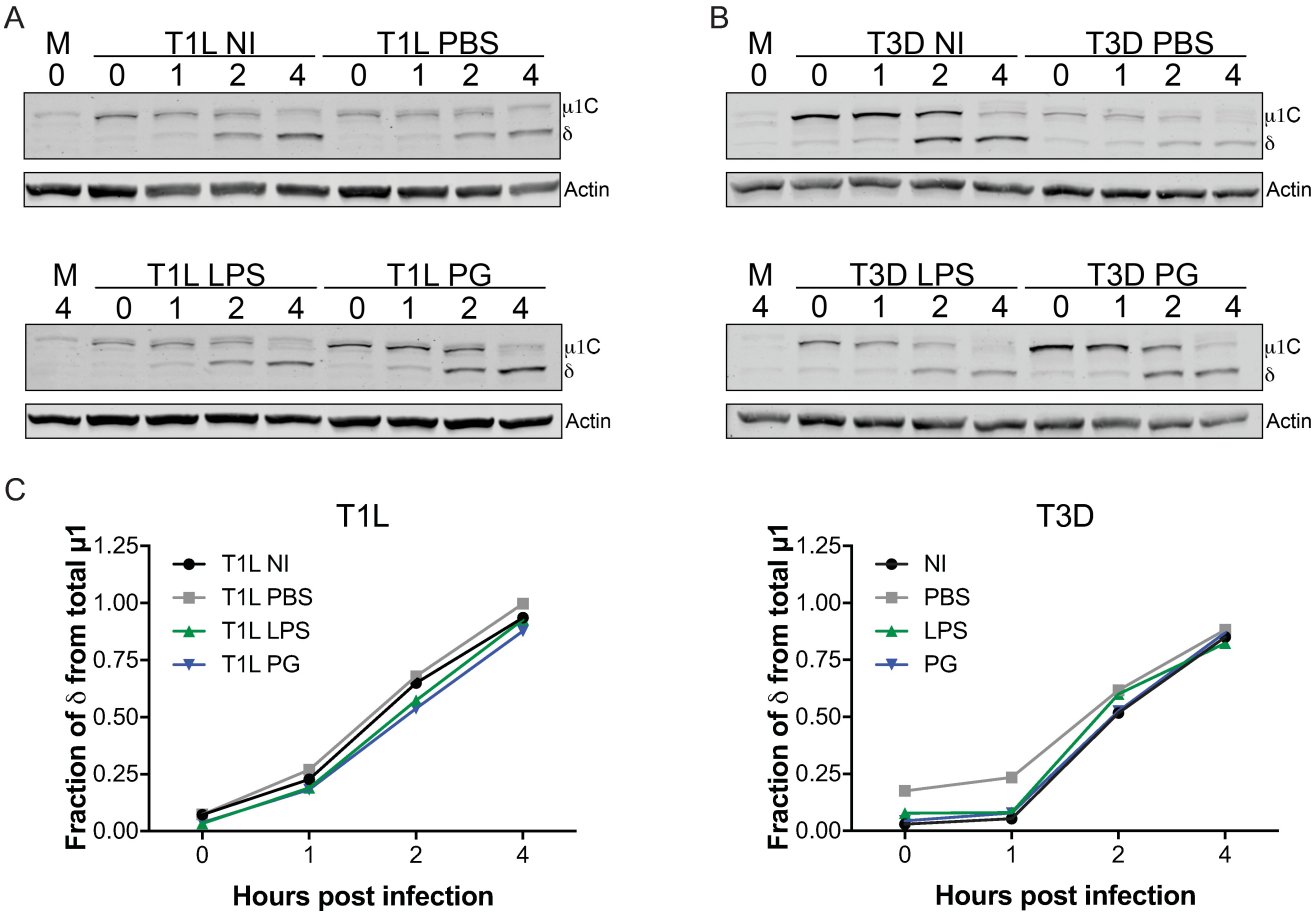
Reovirus cell entry disassembly kinetics are not altered by lipopolysaccharide or peptidoglycan. Reovirus (A) T1L and (B) T3D were incubated not incubated (NI) or incubated in PBS, 10 μg/ml LPS, or 20 μg/ml PG for 2 h at room temperature. Virus was adsorbed for 1 h on chilled HeLa cells, incubated at 37°C, whole cell lysates were prepared at times shown, resolved by SDS-PAGE, and immunoblotted with reovirus-specific and actin antibodies. Position of reovirus μ1C and δ are noted. M = mock. (C) Densitometry analysis of immunoblots shown as the fraction of δ from total μ1 (μ1c+δ).

During cell entry reovirus virions must access acidified endosomes to undergo cathepsin-mediated proteolytic disassembly [26, 30, 53]. During virion disassembly, outer capsid protein σ3 is removed and viral capsid protein μ1 is cleaved into viral fragments that mediate endosomal membrane penetration [34–36]. Proteolytic cleavage of μ1 results in a shortened μ1c form as well as resulting fragments, including the δ fragment, which can be visualized by immunoblot to assess cell entry kinetics [31, 43, 54]. To determine if LPS or PG affect cell entry kinetics, T1L and T3D were not incubated, incubated with PBS, LPS, or PG for 2 h at room temperature. Viruses were adsorbed to HeLa cells for 1 h, incubated for 0, 1, 2, and 4 h post infection, and immunoblotted using reovirus-specific antisera (Fig 9). The cell entry kinetics of non-incubated T1L and T3D were similar, with significant levels of the δ fragment (over 50%) present at 2 h post infection (Fig 9). Incubation of either virus in PBS resulted in an overall decrease in μ1c and δ fragment levels detected at 0 h, with a greater loss of intensity observed with T3D (Fig 9B) than T1L (Fig 9A). This correlates with T1L being more thermostable and having greater infectivity following environmental insult than T3D. Incubation of T1L or T3D with LPS or PG increased the absolute levels of μ1c and δ fragments detected at all time points compared to PBS, but the overall cell entry kinetics were not altered (Fig 9C). The higher levels of μ1c and δ fragments are concordant with LPS and PG enhancing reovirus attachment and infectivity. These indicate that LPS and PG alter reovirus infection through enhancing the stability of the virus, which translates into more virus attaching to cells and an increase in the amount of virus that is taken up by cells for infection.

### LPS and PG do not alter the number of viral attachment fibers on the reovirus particle

**Fig 10:**
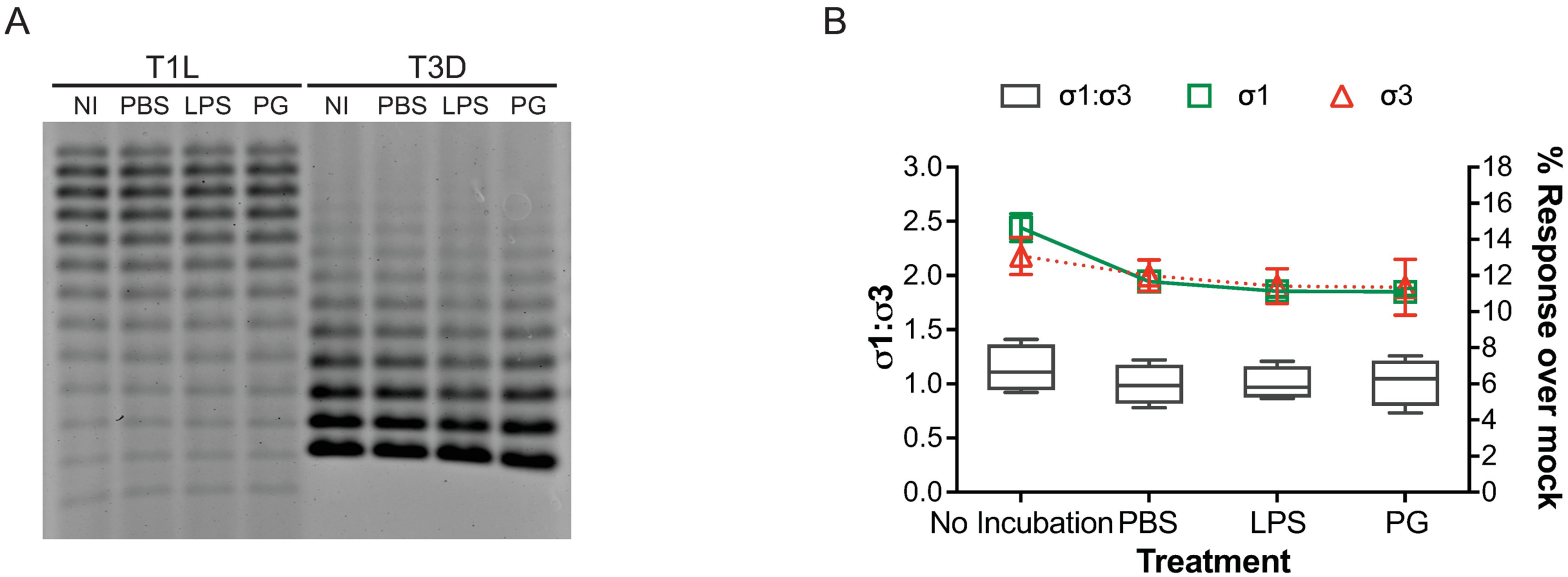
Levels of reovirus attachment fiber σ1 and structural protein σ3 on reovirus are unaltered by environmental assault. Reovirus T1L and T3D were not incubated (NI) or incubated with PBS, 10 μg/ml LPS, or 20 μg/ml PG. (A) The number of σ1 trimers on virions was assessed by separating 1×10^11^ particles by gel electrophoresis. (B) Immunlon high binding plates coated with 5 μg/ml 5C6 (anti σ1) or 2 μg/ml 10C1 (anti σ3) were adsorbed with 5×10^9^ particles of reovirus for 2 h at room temperature. Plates were visualized by indirect immunofluorescence with reovirus-specific antiserum. Data are shown as mean fluorescence intensity over mock of σ1 (green squares) or σ3 (red triangles) for quadruplicate independent experiments. Error bars are SEM from the mean.

The data presented suggests that reovirus loss of infectivity correlates with an impairment in reovirus attachment to cells. The reovirus attachment fiber σ1 is a flexible and filamentous molecule that mediates attachment of the virus with cell-surface carbohydrates and JAM-A [21, 55, 56]. Virions have between 0 and 12 σ1 trimers with no observable difference in infectivity between virions that have 3 or more σ1 trimers [57]. To determine if the observed decrease in infectivity during environmental insult is due to a loss in the number of σ1 molecules on virions and if incubation with LPS or PG affects the number of σ1 molecules on a virion, T1L and T3D reovirus were not incubated, incubated in PBS, LPS, or PG for 2 h at room temperature, and separated by gel electrophoresis (Fig 10A) [57]. T1L and T3D virions had distinct electrophoretic patterns, with T1L virions having on average more σ1 molecules than T3D virions. Incubation of T1L and T3D with PBS, LPS, or PG did not significantly alter the number of σ1 molecules on the virus. These data suggest that environmental stress and incubation of reovirus with LPS or PG does not alter the number of σ1 molecules on virions.

To further assess the integrity of virions following environmental insult, the overall levels of σ1 and major outer capsid protein σ3 were assessed. T1L virions were not incubated, incubated with PBS, LPS, or PG for 2 h at room temperature and assessed for binding to σ1- (5C6) or σ3-specific (10C1) antibodies by fluorescence-linked immunosorbent assay (FLISA) (Fig 10B). Incubation of reovirus with PBS, LPS, or PG caused a small, but not significant, decrease in the levels of σ1 compared to non-incubated virus, corroborating gel electrophoresis data. Incubation of reovirus with PBS, LPS, or PG did not affect overall levels of σ3 on virions. These data indicate that incubation of reovirus with LPS or PG do not affect the overall levels of σ1 and σ3 on the virion. These data also suggest that the σ1 and σ3 antibody-binding epitopes are not altered or masked following environmental assault or by incubation with LPS or PG. Moreover, these data suggest that the decrease in reovirus attachment and infectivity observed following environmental stress are not due to a decrease in the number of σ1 or σ3 molecules on virions.

### LPS and PG do not significantly alter the neutralization efficiency of reovirus-specific antibodies

**Fig 11:**
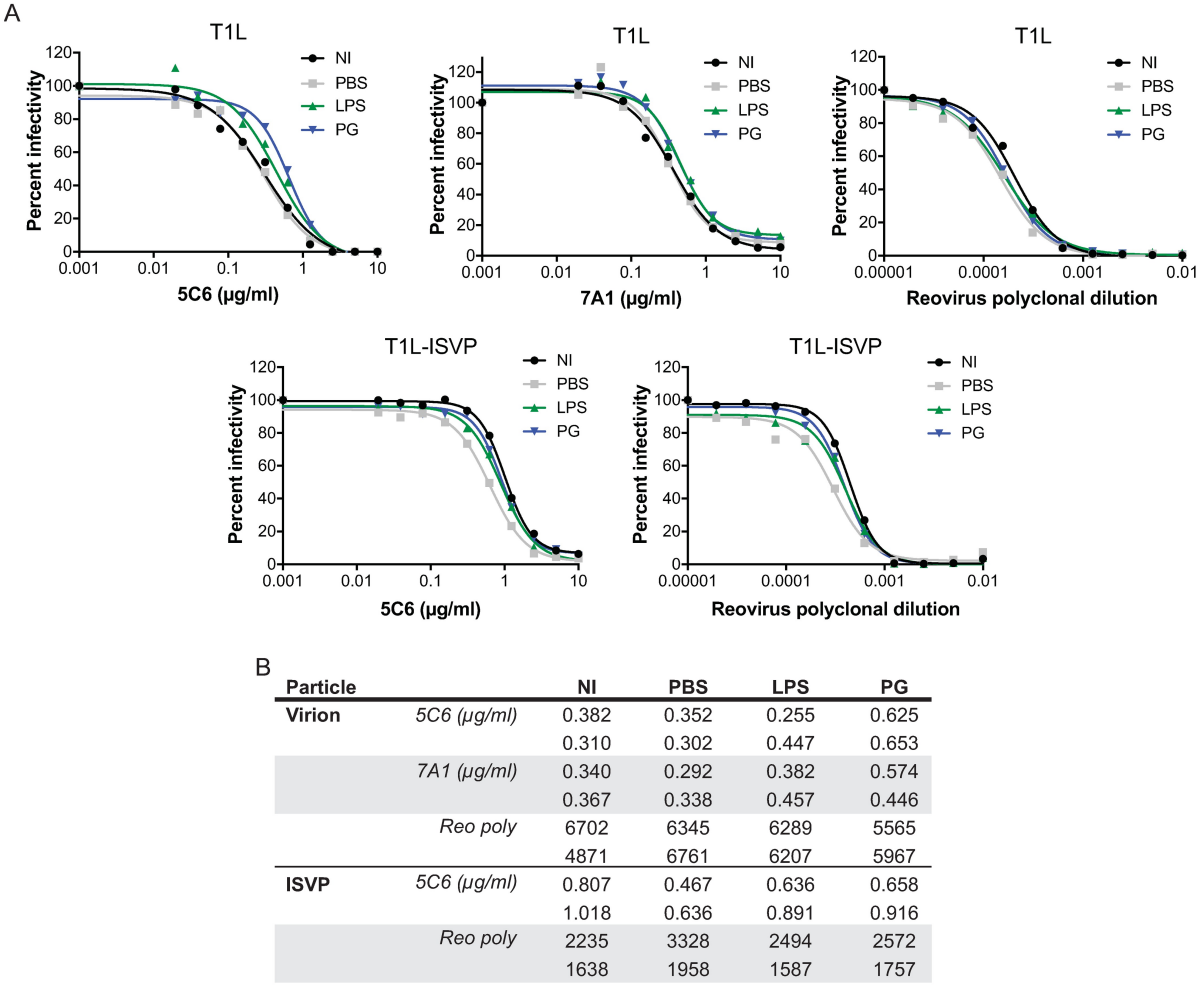
Lipopolysaccharide and peptidoglycan do not significantly impact the neutralization capacity of reovirus-specific antibodies. Reovirus T1L virions (5×10^3^ particles) or ISVPs (1×10^3^ particles) was not incubated (NI) or incubated with PBS, 50 μg/ml LPS, or 50 μg/ml PG for 1 h at room temperature, and incubated with PBS, or increasing levels of anti-σ1 antibody (5C6), anti-σ3 antibody (7A1), or reovirus polyclonal antiserum for 1 h at room temperature. HeLa cells were adsorbed for 1h at room temperature and infectivity was assessed 18 h post infection by indirect immunofluorescence using reovirus-specific antiserum. (A) Results are shown as percent infectivity of duplicate independent experiments with representative graphs. (B) Inhibitory concentration 50 (IC50) are shown in μg/ml for 5C6 and 7A1 and dilution factor for reovirus polyclonal antiserum.

Reovirus infection of mice elicits production of antibodies mainly directed against σ1 [58–61], which impair subsequent rounds of infection, including infection of the gastrointestinal tract [61, 62]. To determine if LPS or PG affect the neutralization efficiency of reovirus-specific neutralizing antibodies, T1L virions and ISVPs were not incubated, incubated with PBS, LPS, or PG for 1 h at room temperature, followed by incubation with increasing concentrations of neutralizing antibody 5C6 (σ1 antibody), 7A1 (σ3 antibody, virions only), or reovirus polyclonal antiserum (Fig 11). Infectivity was assessed by indirect immunofluorescence assay on HeLa cells using reovirus-specific antisera. Incubation of virions with PBS or LPS had minimal effects on the neutralization efficiency of all antibodies tested (Fig 11A). Incubation of virions with PG slightly decreased (less than 2-fold) the neutralization efficiency of both 5C6 and 7A1, but did not affect the efficiency with which reovirus polyclonal antiserum impaired infection compared to non-incubated virus or virus that had been incubated in PBS (Fig 11B). Incubation of ISVPs with PBS slightly enhanced (1.6 fold) the neutralizing efficiency of 5C6 and reovirus polyclonal antiserum, whereas incubation with LPS or PG had negligible effects compared to virus that had not been incubated or PBS-incubated virus. These data suggest that thermal stress and incubation of virions and ISVPs with bacterial envelope components have minimal effects on the efficiency of neutralization by reovirus-specific antibodies. These data also suggest that LPS and PG do not mask the neutralizing epitopes recognized by these antibodies. Data presented in this study show that reovirus particles associate with bacteria through interactions with components of the bacterial envelope. The association with bacterial envelope components enhance virion thermostability that impacts attachment and infectivity of host cells.

## Discussion

Reovirus is a human enteric pathogen that is transmitted via fecal-oral routes and infects intestinal epithelial cells in the gastrointestinal tract [4, 10–14]. Although mostly asymptomatic in humans, reovirus infection affects gut intestinal immune homeostasis, a result of which can be the development of autoimmune diseases like celiac disease [12]. Antibiotic treatment of mice prior to oral infection impairs reovirus infection in the gut and infection-associated pathology in the gastrointestinal tract [38]. However, it is not known how bacteria affect reovirus infection. Data presented here show that Gram negative and Gram positive bacteria promote reovirus infection by enhancing virion thermostability during an environmental insult. The effect on virion thermostability appears largely mediated by bacterial envelope components LPS and PG. These findings parallel those observed with poliovirus, where bacterial LPS enhance virion stability through interactions with the VP1 capsid protein [38, 42]. Interestingly, enhanced reovirus stability by PG and LPS was achieved with over an order of magnitude less than that observed with poliovirus [38, 42]. VP1 interactions with LPS also enhance virus binding to its proteinaceous receptor PVR [42]. In contrast to poliovirus, reovirus attachment was not significantly enhanced by the presence of bacteria or bacterial envelope components. Also, LPS and PG did not affect the efficiency of infection in the presence of antibody against the reovirus proteinaceous receptor JAM-A, suggesting bacterial envelope components do not affect reovirus engagement of JAM-A. These data indicate that bacteria, LPS, and PG enhance reovirus thermostability during environmental stress, providing mechanistic evidence of the role of gut microbiota during intestinal reovirus infection.

The interaction of viruses with host intestinal bacteria is emerging as an evolved strategy enteric viruses use to efficiently infect their host. Clearance of gut bacteria with antibiotics prior to oral infection with poliovirus results in reduced replication and viral-mediated pathogenesis [38]. Similar to that observed with poliovirus and reovirus [38], ablation of the gut microbiota prior to infection with rotavirus, a leading cause of acute gastroenteritis in young children [6], results in reduced rotavirus infection and diarrhea [39]. However, it is not known how the host microbiota promotes rotavirus infection in the intestine. Human norovirus utilizes a different strategy to use host microbes to successfully infect its host. Norovirus utilizes HBGA glycans to attach and infect eukaryotic cells [63]. Norovirus can attach to bacteria that express HBGA glycans and bacteria expressing H-type HBGA enhance norovirus infection of B cells [40, 64, 65]. It is unclear if the mechanism by which bacterially-expressed HBGA enhances norovirus infection of B cells through mediating attachment or enhancing other aspects of viral replication. The gut microbiota can also enhance virus infection through indirect mechanisms. MMTV, a retrovirus that can be transmitted orally though maternal milk [66], binds LPS through LPS-binding proteins incorporated on the viral envelope [41, 67]. During MMTV infection via oral routes, LPS on MMTV virions promotes interlukin-6 (IL-6) secretion following virus infection of gut dendritic cells or macrophages, which in turn stimulate B cells that secrete IL-10 [41, 67]. Through the immunosuppressive properties of IL-10, MMTV is then able to establish a persistent infection and immunological tolerance [68]. Enteric viruses have evolved diverse mechanisms to take advantage of the microbial environment present in the gut to infect their hosts and disseminate. It is unclear, however, if host microorganisms have also evolved to benefit from the presence of enteric viruses in their gut. Interestingly, some bacteriophages can utilize LPS and glycans to attach to bacterial cells [69]. As such, it is possible that reovirus attachment to bacterial cells may provide protection against bacteriophages.

The gastrointestinal tract forms a complex environment composed of changing chemicals, microorganisms, nutrients, and pH [2, 3]. Different segments of the intestinal tract are colonized by distinct bacterial microbial communities [3]. Anaerobes that process simple sugars are more abundant in the small intestine whereas microbes near the distal intestine process more complex, fibrous compounds [3]. In the large intestine intestinal flow is slower and the need for the breakdown of complex polysaccharides results in a greater concentration and species variety of bacterial microbiota [3, 70]. The mucus layer is thicker in distal than the proximal sections of the intestines [70]. As such, it is possible that the effects of bacteria in mediating virus infection is not only determined by the types of bacteria encountered by viruses, but also by the location within the gastrointestinal tract that viruses encounter the microbiota. Reovirus can infect proximal and distal regions of the intestinal tract, although viral titers can be significantly higher in the ileum of infected animals [19]. It is possible that a mixture of host factors, including availability of susceptible cells and the composition of the microbiota in the ileum, provide a more favorable environment for reovirus infection.

Viruses must strike a balance of virion stability that yields optimal infection of the host by providing stability in changing environments during infection and dissemination while also allowing for efficient disassembly of the virion during cell entry. Enteric viruses are exposed to a variety of environmental insults, including dehydration, host and environmental microorganisms, intestinal proteases, low pH, and temperature changes. Although relatively stable, especially compared to enveloped viruses, reovirus infectivity is negatively affected by environmental exposure (Fig 1, S1 Fig and [45, 46]). Reovirus capsid stability can affect the efficiency of viral dissemination within littermates [71] and serotype-specific differences in thermostability, whereby T1L is more thermostable than T2J and T3D, have been genetically linked to the S4 gene segment, which encodes σ3 [72]. The residues in T1L σ3 and the exact mechanisms that govern virion stability are not known.

Why reovirus virions and ISVPs lose infectivity when exposed to environmental stress is unclear. Data shown here and that from other groups [45] show that virions are more stable than ISVPs at temperatures ranging from 23°C to 54°C. Paradoxically, reovirus virions and ISVPs have slightly enhanced stability as temperature increases from 23°C to 37°C. Increased stability at these temperature ranges has been observed [45]. Viral capsids of nonenveloped viruses undergo reversible changes at physiological temperatures called breathing [73]. The changing thermostability of the virus at these temperatures may reflect conformational changes in the viral particle that impact the efficiency with which the particle can engage receptors on the host cell. Suggestive of changes on the viral particle at temperatures between 23°C to 37°C our findings showing the protective effects of PG and LPS on virions and ISVPs being more robust at higher temperatures. Interestingly, viral particles were also not observed to aggregate on the surface of cells, suggesting that bacteria and bacterial envelope components do not enhance stability by inducing the aggregation of viral particles. It is possible that bacteria and bacterial envelope components enhance virion thermostability by reducing viral particle aggregation through a decrease in capsid breathing. These non-aggregated viral particles may be more efficient at engaging cell surface molecules needed for attachment to host cells. BSA, which stabilizes proteins through interactions with hydrophobic regions [48, 49], and other molecules that enhance virion stability, may also do so by reducing viral particle aggregation.

Reovirus attachment is largely mediated by reovirus attachment fiber σ1, though μ1 can affect the efficiency of engagement of host cells [74]. Exposure of reovirus to environmental stress did not affect the overall levels of σ1 or σ3 present in virions, regardless of serotype. These data suggest that an overall loss of the attachment fiber or σ3 are not responsible for the loss of infectivity observed following environmental insult. ISVPs lack viral structural protein σ3, indicating that in the context of ISVPs stability involves viral components other than σ3. It is possible that although σ1 is not lost during environmental stress, conformational changes in σ1 or other capsid components translate to impaired engagement of cell-surface receptors. Incubation of virions in PBS did not affect antibody recognition of σ1, suggesting that at least the antibody recognitions sites within these proteins are not altered when infectivity is impaired. Identifying the viral components affected by environmental insult are likely to inform studies of how non-enveloped viruses regulate capsid stability to thrive in changing environments.

Gram positive and Gram negative bacteria, as well as LPS and PG, enhance reovirus virion stability through steps involved in attachment to host cells. The association of reovirus with bacterial components or bacteria did not cause apparent changes in the overall structure of the particles (Figs 2 and 9). Incubation of reovirus with *E*. *coli* had more robust effects on virion stability than LPS alone, suggesting that another component on the Gram negative bacterial cell or the context in which LPS is presented on a bacterial cell provides enhanced stability to the virus. Detoxified LPS had similar effects on virus stability as full-length LPS, suggesting the lipid A motif in LPS does not mediate the interaction with the virus. In contrast, the effects of purified PG on virus stability were similar to those observed when virus was incubated with *B*. *subtilis*. Enhanced virion stability is not merely due to the presence of a macromolecular object as 3 μm polystyrene beads did not enhance virion thermostability. LPS and PG mediated enhancement of thermostability of both virions and ISVPs, suggesting that they engage a viral structural protein other than μ1 or σ3, as both of these viral proteins are either cleaved (μ1) or not present (σ3) on ISVPs. Identifying the sites on the reovirus virion responsible for LPS and PG binding will be important to determine the degree of conservation of this property across reovirus isolates and to better define the contribution of bacterial envelope components to reovirus replication.

Although bacterial cells, LPS, and PG enhanced reovirus virion stability of both T1L and T3D, we identified polysaccharides that differentially affect the thermostability of these reovirus serotypes. While lipoteichoic acid enhanced T3D stability, it did not impact T1L. Also, chitin had a modest effect on T3D virion stability, while not affecting T1L thermostability. These data suggest that in the context of Gram positive bacteria, T1L and T3D may associate bacterial cells through different components. T1L through PG in a GlcNAc-independent manner, whereas T3D engages the bacterial envelope through GlcNAc sugars on PG and also lipoteichoic acid. Importantly, sucrose did not enhance thermostability, indicating that there is specificity to the contact sites between virions and polysaccharides. The residues within T1L and T3D that mediate the interactions with bacterial cells and polysaccharides remain to be identified. Also of interest, mucin impacted virion thermostability as much as PG in both T1L and T3D. Mucin is a major component of the gastrointestinal tract [2, 3]. It remains to be determined if in the context of the gut, reovirus toggles between bacterial cells and mucin overlaying the gut epithelium to achieve enhanced stability and infectivity and how does the presence or absence of mucin in the gut affect infection.

In addition to binding to its proteinaceous receptor JAM-A, reovirus binds cell surface carbohydrates on eukaryotic cells through its attachment fiber σ1 [20, 21]. Cell entry kinetics were not affected by reovirus incubation with LPS or PG, suggesting that reovirus enters cells by similar endocytic pathways in the presence or absence of bacterial envelope components. It is possible that *in vivo* reovirus engages LPS or PG on the bacterial cell surface, promoting virion stability while also allowing the virus to remain attached to the bacterial cell until the virus can interact with a proteinaceous receptor on a gut epithelial cell or be taken up by an intestinal M cell. In support of this model, incubation of virus bound to *B*. *subtilis* on HeLa cells resulted in virus transferring to HeLa cells with little, if any, virus still attached on the bacteria. Also, incubation of *B*. *subtilis* containing reovirus did not result in the internalization of bacteria by HeLa cells. These data suggest that reovirus is loosely attached to bacterial cells and that the virus can readily exchange bacterial cell binding for eukaryotic cell binding. It is unclear if reovirus tropism in the intestine is altered in the presence or absence of intestinal microbiota.

The interaction of LPS and PG did not significantly affect the neutralization efficacy of two monoclonal antibodies or reovirus polyclonal antiserum. Incubation of virions and ISVPs with PG caused in a small decrease in the neutralization efficiency of a reovirus neutralizing antibodies 5C6 and 7A1. 5C6 targets the σ1 globular domain and is thought to neutralize reovirus infection by affecting virus engagement of cell surface carbohydrate and binding to JAM-A through steric hindrance [75]. 7A1 targets the σ3 major outer capsid protein [76]. The small decreases in the neutralizing efficacy of 5C6 and 7A1 may merely reflect epitopes being less accessible due to the presence of LPS or PG associating with the viral particle. It is unclear if the presence of intestinal bacteria impact the neutralization efficiency or anti-reovirus antibodies *in vivo*. In the gastrointestinal tract, IgA play an important role in the control of enteric viruses [77]. To date, no reovirus-specific IgA monoclonal antibodies have been isolated.

Our study highlights how the interplay between different microorganisms that inhabit a shared environment can affect the outcome of infection. Enteric viruses can use the host microbiota to their advantage during gastrointestinal infections. However, it remains largely unclear how the host microbiota is affected during acute viral infections. It also is intriguing that prokaryotic and eukaryotic viruses have evolved to utilize similar cell-surface molecules to engage bacteria. Data presented here provide evidence that the interaction of viruses with host microorganisms can be beneficial the infectious agent through enhanced biophysical properties of the virion that translates in enhanced infectivity. It remains to be determined if directly altering the composition of the host microbiota can be used as a treatment regimen or to prevent enteric virus-driven disease or if treatment of patients with antibiotics after the onset of disease can alleviate symptoms or help resolve the infection.

## Materials and methods

### Cells, viruses, bacteria, chemicals and antibodies

CCL2 HeLa and Caco2 cells (both from gift from Carolyn Coyne, University of Pittsburgh) were grown in Dulbecco’s Modified Eagle’s Medium (DMEM) supplemented with 10% fetal bovine serum (FBS) (Life Technologies), 2 mM L-glutamine (Life Technologies), 0.11 mg Sodium Pyruvate (Life Technologies), 100 U penicillin per ml (Life Technologies), 100 μg streptomycin per ml (Life Technologies), and non-essential amino acid solution (Life Technologies). Spinner-adapted L929 cells (gift from Terry Dermody, University of Pittsburgh) were grown in Joklik’s modified MEM with 5% FBS, 2 mM L- glutamine, penicillin, streptomycin, and 0.25 mg per ml amphotericin B (Life Technologies).

Reovirus recombinant strains T1L and T3D working stocks were prepared following rescue with reovirus cDNAs in BHK-T7 cells (gift from Terry Dermody, University of Pittsburgh) [78], followed by plaque purification, and passage in L929 cells (dx.doi.org/10.17504/protocols.io.kghctt6) [79]. Purified virions were prepared using second-passage L929 cell lysate stocks. Virus was purified from infected cell lysates by Vertrel XF (TMC Industries Inc.) extraction and CsCl gradient centrifugation as described (dx.doi.org/10.17504/protocols.io.kqqcvvw) [80]. The band corresponding to the density of reovirus particles (1.36 g/cm^3^) was collected and dialyzed exhaustively against virion storage buffer (150 mM NaCl, 15 mM MgCl_2_, 10 mM Tris-HCl [pH 7.4]). Reovirus particle concentration was determined from the equivalence of 1 unit of optical density at 260 nm to 2.1×10^12^ particles [81]. Viral titers were determined by plaque assay using L929 cells [79]. Reovirus virions were labeled with succinimidyl ester Alexa Fluor 488 (A488) or 633 (A633) (Life Technologies) as described (dx.doi.org/10.17504/protocols.io.kqhcvt6) [30, 31]. T1L ISVPs were generated by treating particles with α-chymotrypsin (Sigma) as described [44]. ISVPs were generated by incubating virions with α-chymotrypsin for 20 min.

Reovirus polyclonal rabbit antiserum raised against reovirus strains T1L and T3D was purified as described [79]. Reovirus σ1-specific monoclonal antibody 5C6 and reovirus σ3-specific monoclonal antibodies 10C1 and 7A1 [76] (gifts from Terry Dermody, University of Pittsburgh). JAM-A-specific monoclonal antibody J10.4 (provided by Charles Parkos, University of Michigan). Actin-specific antibody (C-11, Santa Cruz Biotechnology). Secondary antibodies goat anti-Rabbit IRDye 800, donkey anti-goat IRDye 800, and Sapphire700 (LI-COR Biosciences) and goat anti-rabbit A488 (Life Technologies).

*Escherichia coli* (*E*. *coli*) strain K12 (gift from Bruce Levin, Emory University) and *Bacillus subtilis* (*B*. *subtilis*) strain PY79 (gift from Dan Kearns, Indiana University) were grown in LB Miller broth at 37°C. Bacterial lipopolysaccharide (LPS) from *E*. *coli* strain O111:B4 (Sigma and Cell Signaling Technology, detoxified LPS (dLPS) from *E*. *coli* strain O111:B4 (Sigma), and peptidoglycan (PG) from *B*. *subtilis* (Sigma). Lipoteichoic acid from *B*. *subtilis*, mucin from porcine stomach, chitin from shrimp shells, mannan from *Saccharomyces cerevisiae*, and 3 μm polystyrene beads (Sigma). Sucrose (Fisher Chemical), nonfat dry milk (Apex Bioresearch Products), and bovine serum albumin (BSA, Hyclone).

### *B*. *subtilis* expressing mCherry

Generation of *B*. *subtilis* DK400 expressing mCherry. To generate the IPTG-inducible mCherry construct *amyE*::*P*_*hyspank*_*-mCherry*, the *mCherry* gene was amplified from plasmid pDR201 (generous gift of David Rudner, Harvard Medical School) using primers 3224 (aggagaagcttacataaggaggaactactatgg) and 3225 (ctcctgcatgcgtaacatcagagattttgagacttcgaa). The amplicon was digested with HinDIII/SphI and into the HinDIII/SphI sites of pDR111 (generous gift of David Rudner, Harvard Medical School), containing a spectinomycin resistance cassette and polylinker between the arms of the *amyE* gene, to generate plasmid pEV6. The pEV6 plasmid was transformed and integrated into the *amyE* locus of the competent *B*. *subtilis* strain DS2569 [82]. The *amyE* intregant was transduced into *B*. *subtilis* strain 3610 by SPP1-mediated generalized transduction to generate strain DK400 [83].

*B*. *subtilis* DK400 expressing mCherry was grown in LB Miller broth with 1 mM IPTG (FisherBioReagents). Colony forming units (CFU) were determined through serial dilutions of a bacterial culture grown overnight on Luria Broth (LB) Miller-agar (Life Technologies) plates.

### Environmental insult

Reovirus was incubated in phosphate buffered saline (PBS, Cellgro) in the absence or presence of bacteria at equal CFU-to-particles or less or LPS, PG, or indicated reagents at concentrations of 1, 10, 20, 50, or 100 μg/ml for times indicated at 4°C, room temperature (23°C), 28°C, or 37°C with agitation. Reovirus incubated in PBS but kept on ice served as the no incubation control. Reovirus and bacteria, LPS, PG, or component mixture was adsorbed as described below.

Bacterial cultures of *E*. *coli* and *B*. *subtilis* for environmental assault were started from a fresh isolated colony grown in LB Miller-agar plates and grown overnight at 37°C. The bacterial culture was transferred to fresh LB and grown for 4 h at 37°C with shaking. Bacterial cells were pelleted, resuspended in PBS to 45 ml at 0.3 OD 600, and UV irradiated (302 nm wavelength) for 30 min. UV inactivation was confirmed by plating bacteria on LB Miller-agar plates and grown overnight at 37°C. Bacteria were centrifuged and resuspended in PBS to 1/50^th^ the initial culture volume before use in environmental assault.

### Reovirus infectivity assay

Reovirus infectivity was assessed by indirect immunofluorescence (dx.doi.org/10.17504/protocols.io.kqicvue) [84]. Cells were adsorbed with 5×10^3^ particles per cell (virions) or 1×10^3^ particles per cell (ISVPs) for 1 h at room temperature, unbound virus was removed, and incubated for 18 h at 37°C. Cells were fixed with ice-cold methanol and stained with reovirus-specific antiserum (1:1000) and goat anti-rabbit IRDye 800 (1:1000), DRAQ5 cell stain (1:10,000, Cell Signaling Technology), and Sapphire700 (1:1000). Immunofluorescence was detected using a LI-COR Odyssey infrared imaging system (LI-COR Biosciences), and infectivity was quantified using In-Cell Western feature of the Odyssey software suite. For ammonium chloride infectivity assays, cells were incubated with complete medium containing 20 mM ammonium chloride (AC, Fisher Scientific) for 1 h at 37°C prior to adsorption. Cells were supplemented with complete media containing 20 mM AC following adsorption and incubated for 18 h at 37°C. Caco2 infectivity was assessed by staining cells for reovirus antigen with reovirus-specific polyclonal antiserum and goat anti-rabbit A488 (1:1000) and cells with diamidino-2-phenylindole (DAPI, Life Technologies, 1:10,000). Immunofluorescence was detected using a Lionheart FX Automated Microscope (Biotek) with a 4x-PLFL phase objective (NA 0.13), and infectivity was determined by calculating percent infectivity (reovirus positive cells/DAPI positive cells) using Gen5 software (Biotek).

### Flow cytometric analysis of cell-surface reovirus

Cells were incubated at 4°C for 1 h before infection in PBS, or PBS containing 10 μg/ml of JAM-A monoclonal antibody. Cells were adsorbed with 5×10^3^ particles per cell of A633-labeled labeled T1L or T3D for 1 h at room temperature. Cells were detached with Cellstripper (Cellgro) for 15 min at 37°C, quenched and washed with PBS containing 2% FBS, fixed in 1% EM-grade paraformaldehyde (Electron Microscopy Sciences). Mean fluorescence intensity was assessed using a BD LSRII flow cytometer and quantified using FlowJo software.

### Negative stain electron microscopy

Overnight *E*. *coli* and *B*. *subtilis* cultures were washed with PBS and resuspended in 1/10^th^ volume in PBS as the original culture. Bacteria were incubated with reovirus T1L and T3D at an MOI of 1×10^3^ particles per bacterial cell for 2 h at room temperature with agitation. Samples were pelleted and resuspended in Tris- or Phosphate Buffered Saline pH 7.5 (TBS or PBS). Bacteria and virus mixtures were added to 400-mesh carbon-coated copper grids and then stained with 1% phosphotungstic acid (PTA) before imaging using a JEOL JEM-1400 transmission electron microscope (JEOL) equipped with a Gatan US1000 CCD camera (Gatan).

### Fluorescence confocal microscopy

Overnight cultures of *B*. *subtilis* in LB broth spiked with 1 mM IPTG were washed with PBS, resuspended in 1/10^th^ the volume in PBS as the original culture. A488-labeled T1L and T3D were incubated with bacterial suspension at an MOI of 1×10^3^ particles per bacterial cell for 2 h at room temperature with agitation. Bacteria-virus mixture was pelleted, gently resuspended in PBS, and placed on glass slides with equal volume Aqua Poly/Mount (Polyscienes Inc.). Bacteria and virus were imaged by confocal microscopy using an Olympus IX81 laser-scanning confocal microscope using a 60× PlanApo N oil objective with a 1.42 numerical aperture (NA). Pinhole size was the same for all fluors. Single sections of 0.44 μm thickness from a Z-stack are presented.

CCL2 HeLa plated on glass coverslips were incubated with media containing 1 μM CellTracker Blue CMHC dye (Life Technologies) for 30 min at 37°C. Cells were adsorbed for 1 h at room temperature with 5×10^3^ particles of A488-labeled T1L or T3D or adsorbed with A488-labeled T1L and T3D that was incubated with PBS or mCherry *B*. *subtilis* for 2 h at room temperature with agitation. The inoculum was removed, cells were washed with PBS, and placed on glass slides with Aqua Poly/Mount. Images were captured by confocal microscopy as described above.

Whole images were only adjusted for brightness and contrast.

### Reovirus disassembly kinetics

CCL2 HeLa cells were incubated at 4°C for 1 h, adsorbed for 1 h at 4°C with T1L or T3D at an MOI of 1×10^4^ particles per cell. Cells were washed with cold PBS, pre-warmed complete media was added to cells, and whole-cell lysates were prepared using RIPA buffer (20 mM Tris-HCl [pH 7.5], 150 mM NaCl, 1 mM EDTA, 1% NP-40, 0.1% sodium dodecyl sulfate, 0.1% sodium deoxycholate) that was supplemented with Protease Inhibitor Cocktail (Sigma), Phosphatase Inhibitor Cocktail (Sigma), sodium vanadate (Cell Signaling Technologies), and phenylmethylsulfonyl fluoride (PMSF, Sigma). Whole-cell lysates were resolved by gel electrophoresis using PROTEAN TGX-gels (Bio-rad) and immunoblotted using reovirus-specific polyclonal antiserum (1:1000) and actin (1:1000). Immunoblots were scanned using a LI-COR Odyssey infrared imaging system. The intensity of immunoblot bands was quantified using Image Studio software (LI-COR) and the ratio of the intensity of δ protein to that of total μ1 protein was calculated.

### Sandwich FLISA

2HB Immulon 96-well plates (Nunc) were coated with 5 μg/ml 5C6 or 2 μg/mL 10C1 in PBS overnight at 4°C. Wells were blocked with 2% BSA in PBS for 90 min at room temperature. Plates were washed with PBS, incubated for 90 min at room temperature with 5×10^9^ particles per well of T1L or T3D. Plates were washed with 0.5% Tween-20 in PBS (PBST), incubated with reovirus polyclonal antiserum (1:1000) in 1% BSA in PBS (BSA-PBS) for 90 min at room temperature, washed with PBST, and incubated with goat anti rabbit IRDye 800 (1:1000) in BSA-PBS for 1 h at room temperature. Plates were washed with PBST and scanned using a LI-COR Odyssey infrared imaging system. Raw mean fluorescence intensities were normalized to mock and plotted as percent response or percent infectivity.

### Resolution of reovirus particles

1×10^11^ particles of T1L or T3D were diluted in PBS, mixed with 2X loading dye (10% glycerol, 100 mM Tris-Acetate [pH 7.2], bromophenol blue), and separated by gel electrophoresis at 25 V for 18 h (1% agarose in Tris-Acetate [pH 7.2]). The gel was stained with Colloidal Blue (Novex) according to manufacturer’s instructions and imaged using a LI-COR Odyssey infrared imaging system (dx.doi.org/10.17504/protocols.io.krbcv2n).

### Reovirus neutralization assay

Virions or ISVPS were incubated in the absence or presence of neutralizing antibodies 5C6 (σ1) or 7A1 (σ3) at final concentrations of 0.02, 0.04, 0.08, 0.16, 0.31, 0.62, 1.25 or 2.5, or 10 μg/ml or reovirus polyclonal antiserum at 2-fold dilutions from 1:100 to 1:51,200, for 1 h at room temperature with agitation. Samples were adsorbed on CCL2 HeLa cells for 1 h at room temperature, the inoculum was removed, and cells were incubated with complete media for 18 h at 37°C. Cells were fixed in ice-cold methanol and stained as described in Reovirus Infectivity Assay. IC50s were calculated in GraphPad Prism by calculating a non-linear regression curve fit for log transformed infectivity data. The IC50s were obtained from best fit values from curve.

## Supporting information

S1 Fig Effects of temperature on reovirus virion and ISVP stability. (A) Reovirus T1L and T3D were incubated in PBS at 28°C for indicated times, adsorbed on HeLa cells at an MOI of 5×10^3^ particles/cell, and assessed for infectivity at 18 hpi by indirect immunofluorescence. (B, C, D) Reovirus T1L and T3D ISVPs were incubated in PBS at (B) RT, (C) 28°C, or (D) 37°C for indicated times, adsorbed on HeLa cells at an MOI of 1×10^3^ particles/cell, and assessed for infectivity at 18 hpi by indirect immunofluorescence. Results are expressed as box and whisker plots of percent infectivity (normalized to 0 min) for quadruplicate independent experiments. *, *P* < 0.0005 in comparison to 0 h by one-way ANOVA with Dunnett’s multiple-comparison test.
(TIF)

S2 Fig Lipopolysaccharide and peptidoglycan protect reovirus from loss of attachment and infectivity of colonic epithelial cells. Reovirus T1L and T3D were not incubated, incubated with PBS, detoxified LPS (dLPS), LPS, or PG for 2 h at room temperature. (A) Caco2 cells were adsorbed with A633-labeled reovirus at an MOI of 5×10^3^ particles/cell and assessed for reovirus attachment by flowcytometry. Results are expressed as box and whisker plots of cell surface reovirus as MFI for quadruplicate independent experiments. (B) Caco2 cells were adsorbed at an MOI of 5×10^3^ particles/cell, incubated for 18 h, and scored for infectivity by indirect immunofluorescence. Results as percent infected cells for quadruplicate samples. *, *P* < 0.005 in comparison to PBS by one-way ANOVA with Dunnett’s multiple-comparison test.
(TIF)

S3 Fig Lipopolysaccharide and peptidoglycan do not enhance reovirus infectivity. Reovirus T1L and T3D (A) virions or (B) ISVPs were not incubated, incubated with PBS, LPS, or PG for 2 h at 4°C. HeLa cells were adsorbed with reovirus at an MOI of (A) 5×10^3^ particles/cell with virions or (B) 1×10^3^ particles/cell with ISVPs, incubated for 18 h, and scored for infectivity by indirect immunofluorescence. Results are expressed as box and whisker plots of percent infectivity (normalized to no incubation) for quadruplicate independent experiments.
(TIF)

S4 Fig Lipopolysaccharide and peptidoglycan enhance reovirus thermostability at multiple temperatures. Reovirus T3D (A) virions or (B) ISVPs were not incubated, incubated with PBS, 50 μg/ml LPS, or 50 μg/ml PG for 2 h at RT, 28°C, or 37°C. HeLa cells were adsorbed with reovirus at an MOI of (A) 5×10^3^ particles/cell for virions or (B) 1×10^3^ particles/cell for ISVPs, incubated for 18 h, and scored for infectivity by indirect immunofluorescence. Results are expressed as percent infectivity (normalized to no incubation) for quadruplicate independent experiments. *, *P* < 0.0005 in comparison to PBS by one-way ANOVA with Dunnett’s multiple-comparison test.
(TIF)
