# Supplementary figures and images for: Bacteria and bacterial envelope components enhance mammalian reovirus thermostability
Source: PLoS Pathog. 2017 Dec 6;13(12):e1006768. doi: 10.1371/journal.ppat.1006768 (PMC5734793; doi:10.1371/journal.ppat.1006768)

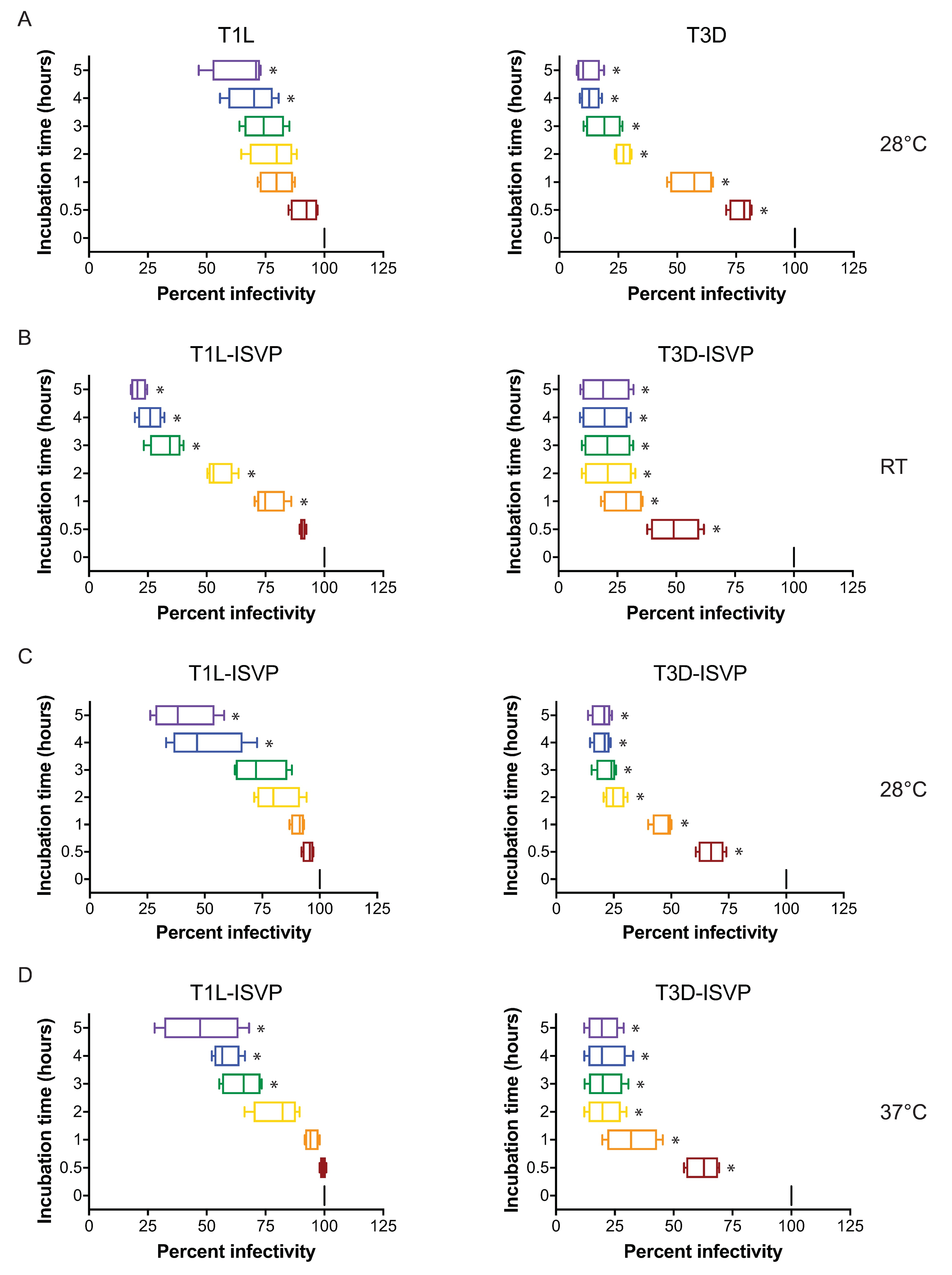

Supplement: S1 Fig — (A) Reovirus T1L and T3D were incubated in PBS at 28°C for indicated times, adsorbed on HeLa cells at an MOI of 5×10^3^ particles/cell, and assessed for infectivity at 18 hpi by indirect immunofluorescence. (B, C, D) Reovirus T1L and T3D ISVPs were incubated in PBS at (B) RT, (C) 28°C, or (D) 37°C for indicated times, adsorbed on HeLa cells at an MOI of 1×10^3^ particles/cell, and assessed for infectivity at 18 hpi by indirect immunofluorescence. Results are expressed as box and whisker plots of percent infectivity (normalized to 0 min) for quadruplicate independent experiments. *, P < 0.0005 in comparison to 0 h by one-way ANOVA with Dunnett’s multiple-comparison test. (TIF) [file ppat.1006768.s001.tif]

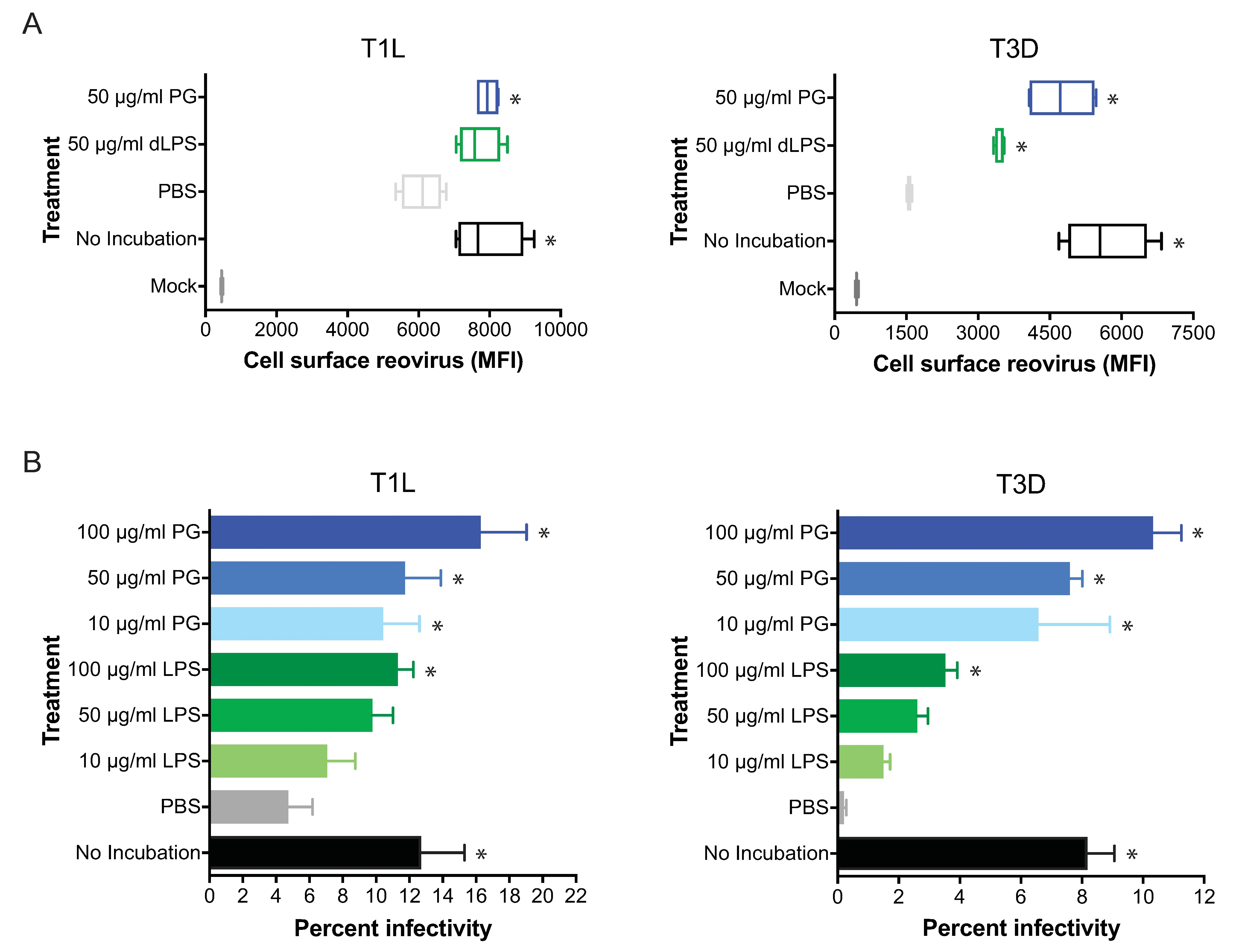

Supplement: S2 Fig — Reovirus T1L and T3D were not incubated, incubated with PBS, detoxified LPS (dLPS), LPS, or PG for 2 h at room temperature. (A) Caco2 cells were adsorbed with A633-labeled reovirus at an MOI of 5×10^3^ particles/cell and assessed for reovirus attachment by flowcytometry. Results are expressed as box and whisker plots of cell surface reovirus as MFI for quadruplicate independent experiments. (B) Caco2 cells were adsorbed at an MOI of 5×10^3^ particles/cell, incubated for 18 h, and scored for infectivity by indirect immunofluorescence. Results as percent infected cells for quadruplicate samples. *, P < 0.005 in comparison to PBS by one-way ANOVA with Dunnett’s multiple-comparison test. (TIF) [file ppat.1006768.s002.tif]

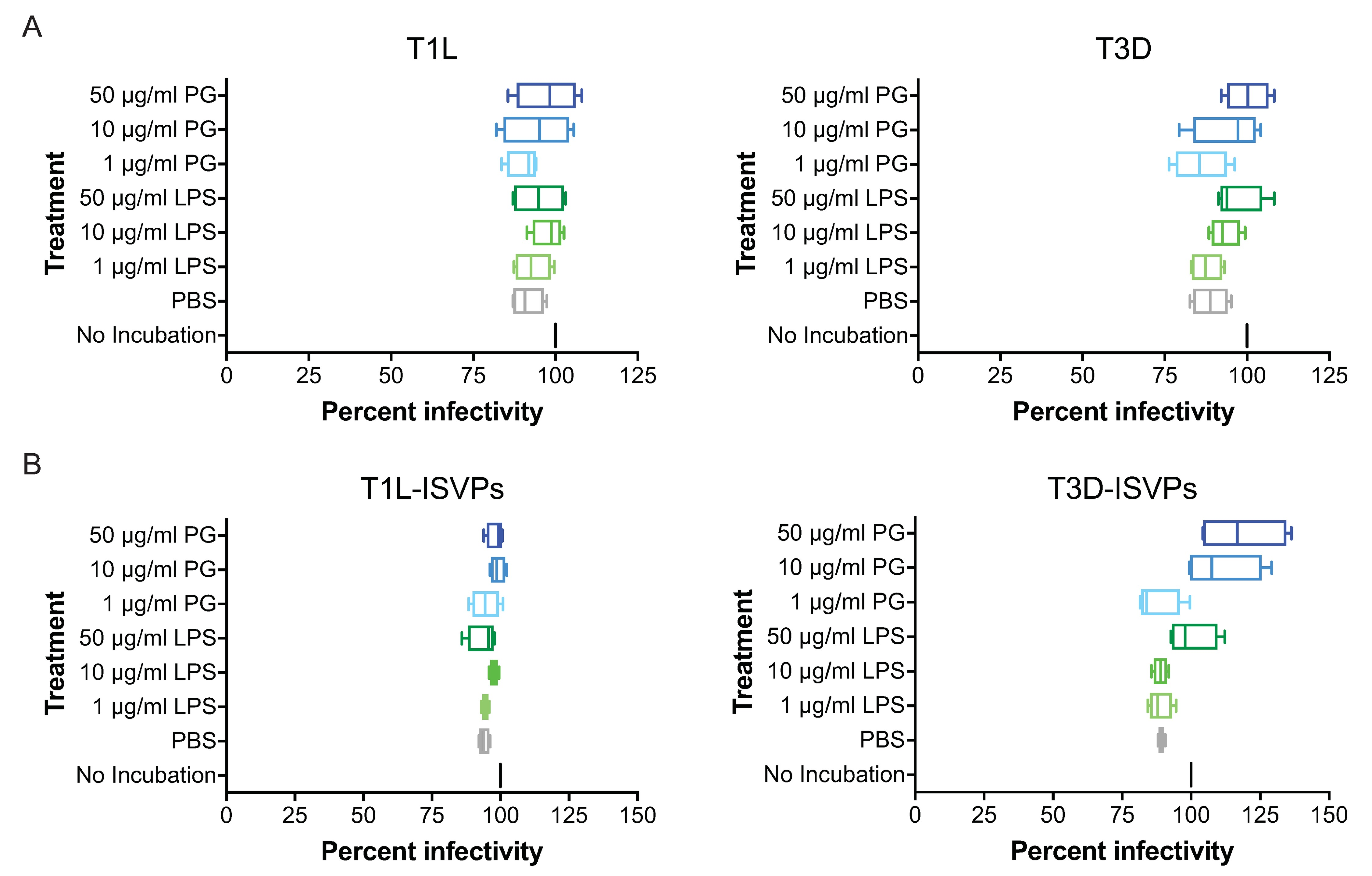

Supplement: S3 Fig — Reovirus T1L and T3D (A) virions or (B) ISVPs were not incubated, incubated with PBS, LPS, or PG for 2 h at 4°C. HeLa cells were adsorbed with reovirus at an MOI of (A) 5×10^3^ particles/cell with virions or (B) 1×10^3^ particles/cell with ISVPs, incubated for 18 h, and scored for infectivity by indirect immunofluorescence. Results are expressed as box and whisker plots of percent infectivity (normalized to no incubation) for quadruplicate independent experiments. (TIF) [file ppat.1006768.s003.tif]

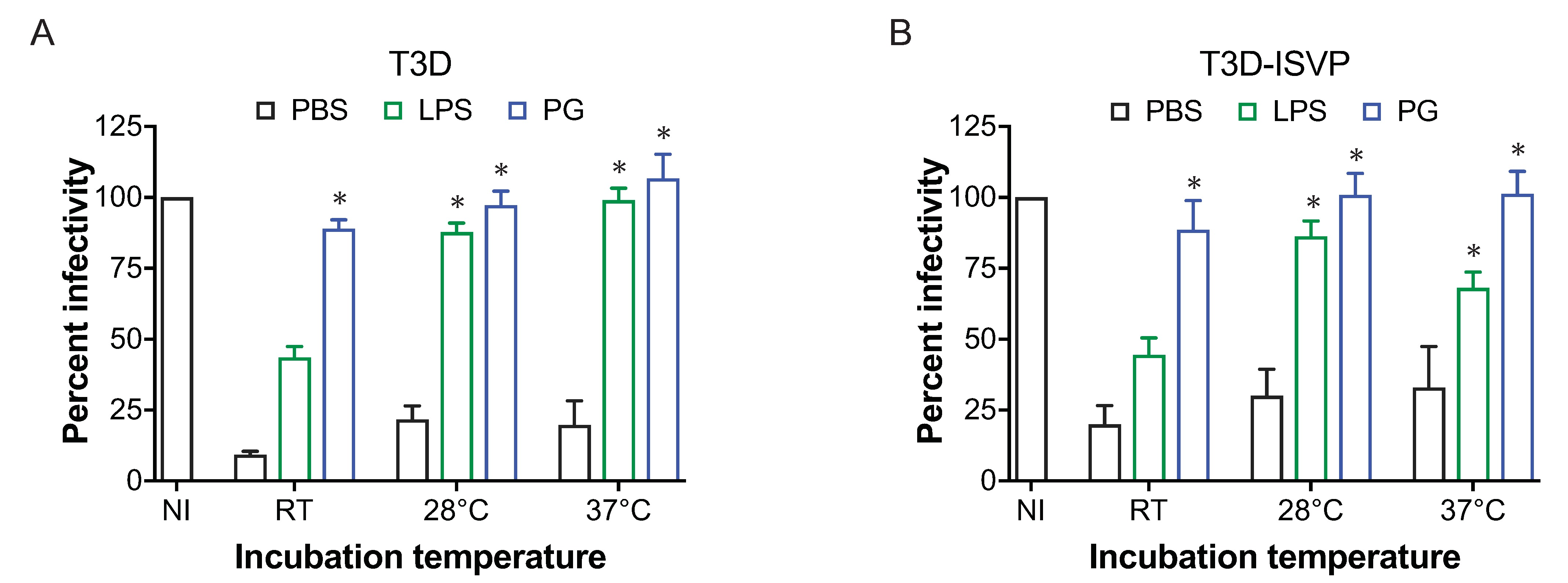

Supplement: S4 Fig — Reovirus T3D (A) virions or (B) ISVPs were not incubated, incubated with PBS, 50 μg/ml LPS, or 50 μg/ml PG for 2 h at RT, 28°C, or 37°C. HeLa cells were adsorbed with reovirus at an MOI of (A) 5×10^3^ particles/cell for virions or (B) 1×10^3^ particles/cell for ISVPs, incubated for 18 h, and scored for infectivity by indirect immunofluorescence. Results are expressed as percent infectivity (normalized to no incubation) for quadruplicate independent experiments. *, P < 0.0005 in comparison to PBS by one-way ANOVA with Dunnett’s multiple-comparison test. (TIF) [file ppat.1006768.s004.tif]
